# FMD-VS: A virtual sensor to index FMD virus scattering

**DOI:** 10.1371/journal.pone.0237961

**Published:** 2020-09-11

**Authors:** Kayoko Takatsuka, Satoshi Sekiguchi, Hisaaki Yamaba, Kentaro Aburada, Masayuki Mukunoki, Naonobu Okazaki

**Affiliations:** 1 Faculty of Engineering, University of Miyazaki, Miyazaki, Japan; 2 Faculty of Agriculture, University of Miyazaki, Miyazaki, Japan; Universidad Veracruzana, MEXICO

## Abstract

Foot-and-mouth disease (FMD) models—analytical models for tracking and analyzing FMD outbreaks—are known as dominant tools for examining the spread of the disease under various conditions and assessing the effectiveness of countermeasures. There has been some remarkable progress in modeling research since the UK epidemic in 2001. Several modeling methods have been introduced, developed, and are still growing. However, in 2010 when a FMD outbreak occurred in the Miyazaki prefecture, a crucial problem reported: Once a regional FMD outbreak occurs, municipal officials in the region must make various day-to-day decisions throughout this period of vulnerability. The deliverables of FMD modeling research in its current state appear insufficient to support the daily judgments required in such cases. FMD model can be an efficient support tool for prevention decisions. It requires being conversant with modeling and its preconditions. Therefore, most municipal officials with no knowledge or experience found full use of the model difficult. Given this limitation, the authors consider methods and systems to support users of FMD models who must make real-time epidemic-related judgments in the infected areas. We propose a virtual sensor, designated “FMD-VS,” to index FMD virus scattering in conditions where there is once a notion of FMD; and (2) shows how we apply the developed FMD-VS technique during an outbreak. In (1), we show our approach to constructing FMD-VS based on the existing FMD model and offer an analysis and evaluation method to assess its performance. We again present the results produced when the technique applied to 2010 infection data from the Miyazaki Prefecture. For (2), we outline the concept of a method that supports the prevention judgment of municipal officials and show how to use FMD-VS.

## 1. Introduction

Foot-and-mouth disease (FMD) is a contagious infection that affects livestock such as cattle and pigs, and wild creatures such as deer and wild boar. Outbreaks in FMD-free countries can lead to enormous economic losses because of direct and indirect reductions in productivity [1–3]. Although we should carry out culling at once upon detection, the infection is undetectable during the incubation period. To clarify this issue, we have provided studies for the preclinical diagnosis of FMD [4–7]. However, the associated research and development are expensive. Although the method of delaying the spread of diseases by vaccination is valid, the issue is that the inoculated animals have to be culled after the inoculation to suppress the spread of the virus (treated to death). Under the international OIE (World Organization for Animal Health) agreement, since it is impossible to identify antibodies formed in the treated animals from antibodies formed by viruses, we have considered inoculated animals as infected. Marker vaccine has been encouraged to identify vaccination from the disease. If the NSP (non-structural protein) antibody is confirmed negative, then inoculated farm animals need not be slaughtered (inoculate to live) [6]. However, the marker remedy was unused in the case of the Miyazaki Prefecture in 2010 and, thus, we could not confirm the reliability of the commercial test kit for the NSP antibody. Lack of an epidemic budget is one reason that the marker vaccine is not more widespread. Even though rapid culling and disposal of carcasses are effective [8,9], securing proper burial sites takes time.

Given these issues, we have perceived analytical models capable of tracking FMD transmission as valid tools for validating the effects of preventive measures and tracing the spread of the disease under various conditions. Models of infection spread range from simple deterministic analytic models to structure, certain stochastic microsimulations [10–13]. We can distinguish the designs based on how they handle time (discrete/continuous), space (spatially explicit/non-spatial), and chance or uncertainty (deterministic/ stochastic) [14]. Another way of classifying models is according to whether they are population-based, individual-based, or a mix of both.

Over the years, we have studied and proposed various types of FMD models and methods [8, 11–13, 15–21]. Modeling research has been active since the 2001 FMD outbreak in the UK. The Keeling model is a prominent example of such research [15]. The Keeling model simulates the infection probability of FMD on a farm unit basis and has been used to analyze the effects of vaccination and preventive culling [9, 16, 17]. In at least one example, we have applied the Keeling model to the case of the 2010 the Miyazaki Prefecture epidemic in Japan [2, 18]. Although not applied to Japan, we introduced a Bayesian Ensemble Approach. It uses the Warwick model [15, 16], a development of the Keeling model, which shows an implementation with a single-model ensemble based on different parameterizations of the model, allowing for a comparison of the effectiveness of various epidemic measures [20]. Also, we recommend a hybrid model that combines a model based on deterministic equations (EBM) for modeling the diffusion of FMD in farms with a probabilistic and spatially explicit, agent-based model (ABM) for modeling the spread between groups [22]. Like the Keeling model [23], this approach is still development.

The 2010 FMD infection in the Miyazaki Prefecture highlighted an important issue. Once regional infections of FMD occur, municipal officials in the region had obligations to make multiple daily judgements. However, the deliverables of current FMD modeling research cannot support the daily decision-making required in the infected area. Staff in charge at the time of the Miyazaki outbreak commented, “The stress was huge. We had to make a wide variety of decisions daily using intuition on matters ranging from things directly connected to the epidemic, such as movement restrictions, vaccinations, and culling, to regional activities such as school, events, and mail delivery.” Many researchers in FMD modeling have the view that providing the model itself suffices to help general decision-making for epidemic prevention. They believe that their purpose is solely to provide an FMD model-based simulation tool, leaving the administrators to analyze simulation results and make decisions for epidemic prevention.

How did such differences in perspective develop? As described in *RAPIDD 2015 in Miyazaki* [24], staff members in charge at the time of the Miyazaki FMD outbreak held a face-to-face meeting with modeling researchers. Based on the interactions that took place at the meeting, the summary of our impression is: Many of the deliverables of FMD modeling research come in the form of simulation tools to verify the spread of the disease under different conditions and to assess the effectiveness of epidemic measures [9, 16, 17, 19, 20]. Using it adequately, it can function as a support tool for epidemics decisions. However, if we use it with no measures, we will only be able to analyze trends, and it cannot be a means to support the judgment of epidemics. Modeling researchers believe they are providing accurate deliverables. However, FMD modeling requires a certain level of familiarity with the preconditions and with the model itself [13]. Therefore, the prefectural officials in charge at the time of the Miyazaki outbreak did their best to use the model for trend analysis, but could not use it to decide—they did not have the requisite experience or knowledge of modeling.

In attempting to address this serious issue, the authors looked to operations control in chemical plants (PSE143seminar, 2018). To operate and control chemical-plants, robustly, modeling, and simulation of thermal energy and fluid performance analysis are required. However, many plants are operated and controlled by operators who do not have modeling or chemical engineering expertise. How is this possible? Experts familiar with modeling and chemical engineering provide explicit instructions. We sought to develop a similarly effective support system for FMD model utilization. Such a support system would enable administrative staff lacking knowledge of FMD modeling and infectious diseases to use the models with minimum stress. In this paper, as an elemental technology, we designed a virtual sensor to help users grasp the extent of virus scattering on individual farms in an infected area. In particular, we created a virtual sensor that we call FMD-VS to monitor FMD virus scattering and index the amount of scattering in situations where FMD already exists.

The virtual sensor we propose simulates a conception in recent control engineering theory known as a soft sensor [25–30]. A soft sensor is a technology realized by software that estimates the volume of a target (such as the concentration of fluid) that is difficult to measure in real-time with a normal (hard) sensor. Intuitively, the virtual sensor works as if we tested the target. In actuality, it is a multivariate relational expression related to the target quantity. We consider the numerical value achieved by the formula calculation as a virtual measurement value.

There are two methods to create a soft sensor, one based on a physical model and the other based on a statistical technique. A soft sensor based on a physical model with a deep understanding of the target process is desirable in the sense that anyone can appreciate and apply it. However, in reality, it is often complex to design a software sensor that can withstand practical use based only on physical models; in such cases, it requires the power of statistical methods fully or partially (partial quotes from [25]).

Adopting the soft sensor theory, we base the desired virtual sensor on an epidemiological model (FMD model) that reflects a deep understanding of FMD spatial transmission. However, conventional soft sensors imply an absolute quantity, such as fluid concentration. In contrast, the extent of scattering virus, the target of FMD-VS speculation, cannot be expressed as a definite quantity. For this reason, we use the value measured by FMD-VS as an index to indicate scattering.

As described above, we propose a pseudo sensor which we call FMD-VS, for estimating and indexing the amount of FMD virus scattering in a way that simulates the conception of a soft sensor (or virtual sensor). As described in the following sections, FMD-VS originates from an existing FMD model. We further show a method for evaluating the performance of the FMD-VS approach and a way to classify factors for improving its performance. We again apply the proposed method to data from the Miyazaki Prefecture in 2010 and show the results. We summarize our conclusions after presenting our ideas relating to implementing the technique.

## 2. Materials and methods

### 2.1 Data

April 20^th^, 2010, an onset of FMD caused by serotype O-virus (topotype Southeast Asia, genotype Mya-98) was confirmed in the Miyazaki Prefecture, in the southern part of Japan. The affected area was among the leading livestock areas of Japan: populated with both cattle and pigs (about 6,600 cattle farms and 400 pig farms). Control projects such as movement restrictions, destruction of animals on affected farms, and strengthening biosecurity measures were carried out at once after the first detection. It developed into a large-scale transmission epidemic despite these actions. A month after they detected the initial infection, they executed an emergency vaccination program using the oil-adjuvant FMD vaccine (O1-Manisa) over a range of 40 km along the north-south axis of the Prefecture. By August 27^th^, 292 farms were affected, and approximately 290,000 animals were destroyed [2], infected, or vaccinated.

To conduct the present study, we used the 2010 infection data from the Miyazaki Prefecture (Appendix) made available by the Agriculture Ministry on its website [31], the only official data for FMD in Japan. The items included are farm type, number of animals, date and time of infection, and date and time of completion of the culling of 292 infected farms. The Appendix provides the latitude and longitude of the 292 farms, as found on Google Earth.

### 2.2 Introduction to FMD-VS

Summary of the essential nature of FMD-VS: if we suspect FMD, detect the atmospheric dispersion of FMD virus and introduce FMD-VS to show the expansion of virus scattering on a farm-by-farm basis.

#### 2.2.1 Calculation formula for the scattering index

We refer to the value gained by FMD-VS to show the degree of FMD virus scattering as the “FMD virus scattering index” or the “scattering index.” We base the formula used to estimate the Keeling model, known as the FMD model. Although not entirely reliable [32], the model has a reasonable and tractable mathematical expression. It simplifies the complicated process of virus transmission between farms and covers between-farms propagation with a deep understanding of the course of an epidemic. By building on the Keeling model, we believe that most practitioners and modelers will use our method of full use. We should note that several model variations have originated from the Keeling model. The actual model developed in our method is the model presented in Hayama et al. [8]. Our model is the same as the Keeling model but has a different formula composition. We use inter-farm transmission parameters to estimate the probability of spatial transmission between farms based on inter-farm distances using estimates from the 2010 Miyazaki Prefecture infection data. The model of Hayama et al. proposes the following elements:

$$P_{inf,i}\left(t\right)=1-exp\left(-\lambda_{i}\left(t\right)\right)$$ (1)

$$\lambda_{i}(t)=\sum_{j\in \inf ections}C_{ij}N_{i}N_{j}h(r_{ij})$$ (2)

$$h\left(r_{ij}\right)=h_{0}\left(1\;+\;r/r_{0}\right){-}^{\alpha }$$ (3)

(“infections” refer to the group of all infected farms)

Here, *P*_*inf*,*i*_*(t)* is the probability that it infects susceptible farm *i* on day *t*, starting from the first time the area identified infection. *N*_*i*,_ which is a component of the main parameter, *λ*, of *P*_*inf*,*i*_*(t)*, is the number of animals at the farm of interest *i*; *N*_*j*_ is the number of infected animals at infected farm *j* affecting farm *i*. *C*_*ij*_ is the probability that farm *i* will be infected by farm *j*. Term *h(r*_*ij*_*)* is the transfer parameter, which depends on the distance *r*_*ij*_ between farms *i* and *j*, but maximum likelihood estimation acquire other parameters *h*_*0*_, *r*_*0*_, and *α* possessed by *C*_*ij*_ and *h(r*_*ij*_*)* based on the infection data. For this reason, *h(r*_*ij*_*)* takes a value that also includes differences in virus species. In this study, we use the parameters estimated by maximum likelihood using the Miyazaki prefecture data shown in Section 2.1. *r*_*0*_ = 0.58, *h*_*0*_ = 0.00074, *α* = 2.47, and if *C*_*ij*_ shows a cow-to-cow transmission probability of 1, the cow-to-pig transmission rate is 0.77. The transmission probability from pig-to-cow is 2.45, and from pig-to-pig is 3.01.

The above FMD model copes with a spatial transmission, in which the wind spreads the viruses. However, direct contact between animals or indirect contact through people or feed by aerosol also spread the FMD virus. Here, we assumed that the distance-based transfer parameters of the FMD model and spatial transmission express the possibility of aerosol infection. We consider this adequate, as the probability of aerosol infection increases as the sites locate closer together.

In this research, we use the formula for *λ* given in (2) to calculate the scattering index. We believe that it suits this formula to that purpose since it comprises observable and measurable indicators of virus scattering, such as the scale of the infected farms in the vicinity (measured in the number of animals) and the distance to the infected farms. In the following discussion, we refer to the model proposed by Hayama et al. as the FMD model. 'Scattering index' is synonymous with '*λ'*.

### 2.3 FMD-VS performance evaluation

#### 2.3.1 Approach

We tested performance based on the accuracy of the measured values with sensors (i.e., hard sensors) and soft sensors. However, FMD-VS measures the amount of scattering virus that absolute quantity cannot determine. Therefore, we cannot test performance with the accuracy of the measurements. Performing FMD-VS, in contrast, will measure its ability to estimate the time at which a random farm changes from susceptible to infectious, based on the scattering index (referred to as “discrimination ability”). We first sought to establish whether our method had actual discrimination ability. By determining the value of the scattering index based on whether the time at which farm is most susceptible to infectivity. To some extent, we can judge this from the shape of the histogram showing the distribution of the scattering index of the time infected. If the histogram is roughly bell-shaped, then FMD-VS can consider having at least some discrimination ability. Multiple irregularities in the histogram with a broad peak imply the difficulty of the most likely timing of a change to infectious.

**Fig 1:**
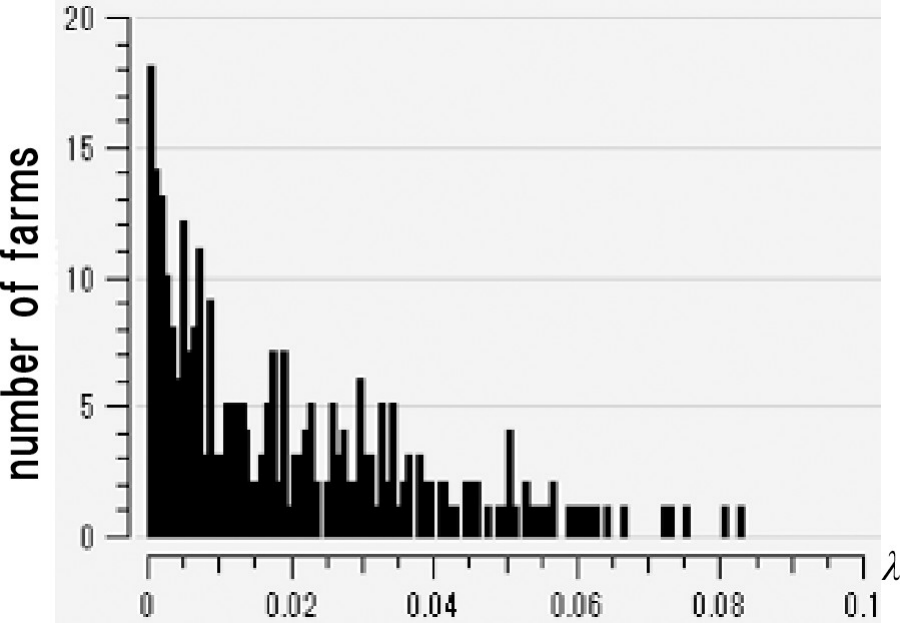
Distribution of *λ* (scattering index) on the day of infection for each farm estimated from the data. The distribution is based on the only official FMD infection data in Japan (2010 data for Miyazaki prefecture). The date on which the individual farms changed to infectivity was estimated from the day of infection indicated by this official data.

*2*.*3*.*1*.*1 Creation of scattering index histogram and cumulative frequency distribution*. Fig 1 is a histogram of the scattering index of the infection date estimated from the data of the 292 farms infected in 2010.

**Fig 2:**
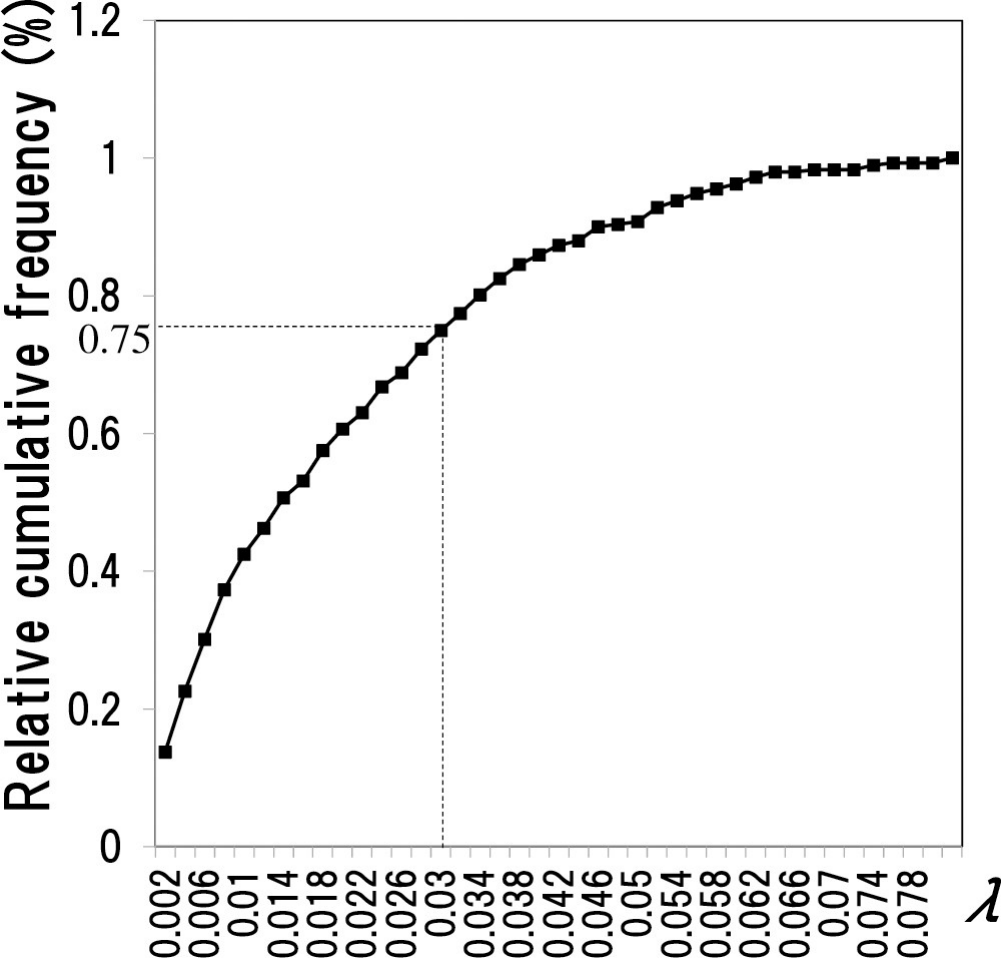
Cumulative relative frequency distribution of *λ* (scattering index) of the day of infection for each farm estimated from data.

The horizontal axis represents *λ*, the scattering index; the vertical axis represents the number of farms. We calculate the scattering index of farm *i* (*i* = 1, …, 292) as follows:

First, we provisionally estimate the date of infection on farm *i* from the 2010 infection data (Appendix). Since the infection date is 7 to 10 days before the onset of FMD, we assume that the infection day is seven days before the infection detection date for convenience. We then clarify the distribution of the infected farms on the time of infection on farm *i*. We estimate the infection day of each farm in the way described above. We can then find the scattering index on the date of infection on a farm *i* using expression (2). We equally determine the scattering index of farm *i* (*i* = 1, …, 292) on the time of infection. Fig 1 is the histogram created from the data; Fig 2 is the cumulative frequency distribution. The horizontal axis shows values for *λ*, the scattering index; the vertical axis is the cumulative relative frequency of the scattering index.

Unfortunately, we found many irregularities in the scattering distribution index (Fig 1) created from the Miyazaki Prefecture data (292 infected farms). The histogram does not show the desired bell-shape. However, Fig 1 does not represent an accurate distribution, as this histogram has two problems: First, the histogram consists of only 292 data points, which we considered insufficient. Many natural phenomena refer to the bell-shaped probability density functions such as normal distribution and Maxwell distribution. We assumed that the scattering index may relate to this shape distribution. However, according to the law of large numbers, a limited number of data points cannot provide an appropriate distribution. The second problem is the uncertainty associated with the date of infection given in the data. These two factors suggest that Fig 1 may not represent the appropriate distribution. To deal with these two problems, we considered a two-part solution: First, to deal with the data insufficiency problem, we conducted cross-validation. Cross-validation is a technique used to confirm statistical estimates derived from data when the data set is relatively small. Cross-validation is a method of repeatedly applying the hold-out method while exchanging train data and test data, and testing the average of the evaluation results of each time [33]. The hold-out method is a general method for evaluating the entire data set by dividing it into training data used to create a model and test data used to test the model. The next problem is the uncertainty of infection. We defined the concept of “around the time of infection” and made it possible to address this with the hold-out evaluation method. We show this more concretely in the next section.

#### 2.3.2 Analysis and evaluation

We developed a statistical method to decide the value of the scattering index at the time that a farm is most susceptible to infectivity. As described above, in this method, cross-validation was used to discuss the small sample size problem. This procedure also provided an effective response to the uncertainty associated with the actual date of infection. By applying our method to the Miyazaki infection data, we found it possible to determine the value of the scattering index at the time of susceptibility.

**Fig 3:**
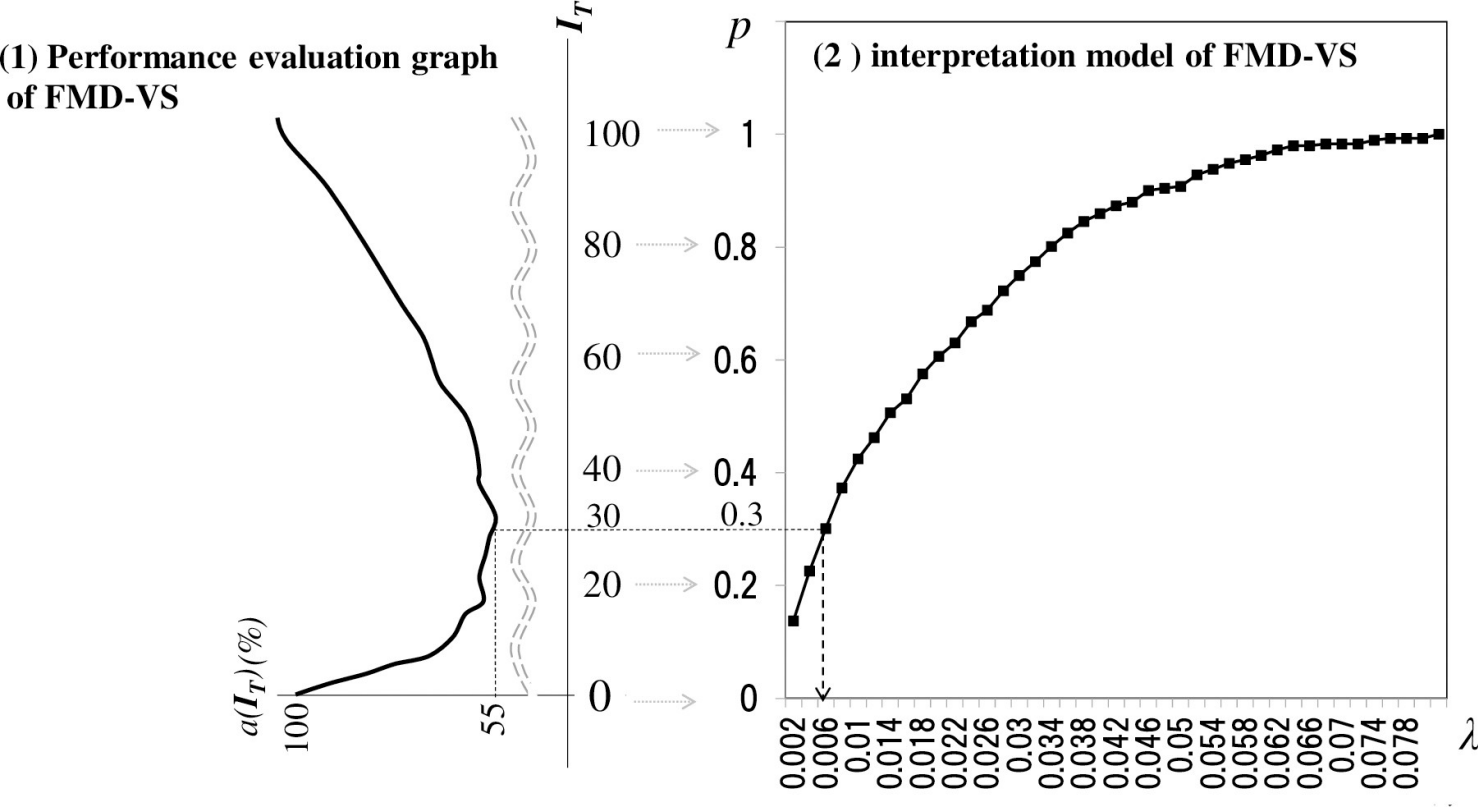
Purpose of the FMD-VS performance evaluation graph. For example, the minimum *a(I*_*T*_*)* value of 55% on the performance evaluation graph associates threshold *I*_*T*_ = 30 → probability *p* = 0.3 → scattering index λ = 0.006. This means that we were able to confirm (from the actual data) that, in 55% of the cases, a farm changed to infectivity when the threshold value of 30 was reached, or, more importantly, at a time when its scattering index first exceeded 0.006.

Our method analyzes and tests the discrimination ability of FMD-VS based on the distribution (histogram) of the scattering index. We repeated the hold-out procedure, exchanging the Train Data and the Test Data for the scattering index of the 292 farms on the time of infection, and tested the results using the mean of the evaluation values. The method is:

1. Model generation for the hold-out method: We classify the 292 scattering indices into Train Data and Test Data, then devise a model for evaluation with the Train Data. We create a histogram of the scattering index (see (Fig 1)) with the Train Data alone. We then create the cumulative relative frequency distribution (see (Fig 2)) and apply it as a model for evaluation. The horizontal axis is *λ* (scattering index); the vertical axis shows the cumulative relative frequency of the scattering index values.
2. Model performance evaluation with the Test Data (preparation): Prepare a performance evaluation graph format like the graph in Fig 3-(1) to display the evaluation results in a visually easy-to-understand form.

The horizontal axis represents the value *I*_*T*_ achieved by normalizing the entire scattering index on the horizontal axis of the model for evaluation (see Fig 3-(2)) to 0 to 100. For example, the scattering index in Fig 3-(1) has a value ranging from 0.002 to 0.078. Because of normalizing to 0–100, *λ* = 0.006 is normalized to 30. We normalize the scattering index *λ* based on the model (Fig 3-(2)) in the following way: For *λ* = 0.006, the cumulative relative frequency is *p* = 0.3; we normalize *λ* by noting 0.3 as a percentage. Thus, the normalization of *λ* = 0.006 becomes *p* * 100 = 30.

3. Performance evaluation of the model with the Test Data: In this step, we select one of the normalized scattering index values *I*_*T*_ *(I*_*T*_ = 1, …, 100) and use the selected *I*_*T*_ value as the threshold value (referred to as the discrimination threshold or threshold). Using this threshold, we then calculate the discriminant ability of the model and plot the result on the performance evaluation graph (Fig 3-(1)). Calculating the discriminating ability means to calculate the discrimination failure rate (strict definition provided later) when we specify infection using *I*_*T*_ as a threshold. For example, suppose we assume that the threshold *I*_*T*_ is 30. As described above, the value of *λ* associated with this *I*_*T*_ is 0.006 (from Fig 3-(1) and (2)). We then look for the number of farms, N, that has never exceeded the *λ* = 0.006 value "around the time of infection day" (described later) and calculate the percentage of such farms relative to the 292 infected farms and label it (*I*_*T*_)'. Thus, (*I*_*T*_)' = (N / 292) * 100. We treat (*I*_*T*_)' as the discrimination failure rate for the case in which we set the threshold value at *I*_*T*_ = 30.
3. Cross-validation of the performance evaluation: Here, we repeat steps 1 to 3, exchanging Train Data and Test Data.

We get the discrimination failure rate (*I*_*T*_)’ for each of *m* repetitions and finally determine the mean value *a*(*I*_*T*_):

$$a\left(I_{T}\right)=\Sigma j=1m\;a\left(I_{T}\right)'j/m$$ (4)

With *I*_*T*_ = 30, the mean discrimination failure rate *a*(*I*_*T*_) is 55; (30, 55) is plotted in Fig 3-(1).

4. Complete the performance evaluation graph: We determine the mean values of the discrimination failure rates for all the threshold values of the normalized scattering index *I*_*T*_ (*I*_*T*_ = 1, …, 100), and plot the results on a graph such as that in Fig 3-(1).

*2*.*3*.*2*.*1 Definition of “around the time of infection”*. We noted in Section 2.2.1 the possibility that the scattering index histogram (Fig 1) on the time of infection for each farm may not represent the correct distribution and raised the suspicion of disease estimated as one cause. Therefore, as a way of addressing this uncertainty issue, we did not specify the expected time of infection to a particular day. We call the several consecutive days, including the time we identify the infection as around the time of infection, and thus assuming the possibility of infection somewhere “around the time of infection" set arbitrarily.

**Fig 4:**
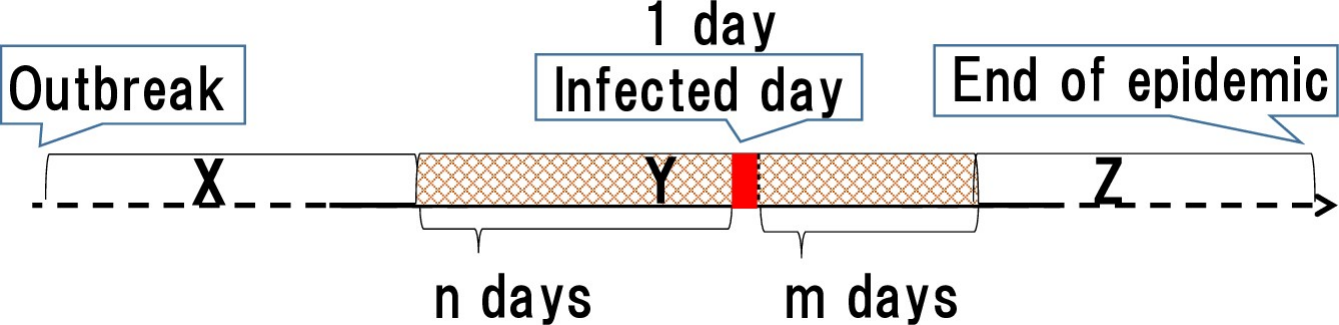
Period including the infected day. “Around the time of infection” indicates the period from several days before to several days after the “day infection was identified” on the infection data. If n is the number of days before the day of infection and m is the number of days after the day of infection, then the period considered to be “around the time of infection" is n+1+m days.

Fig 4 analyzes this idea of "around the time of infection" using a graph of the time series from Outbreak to End of the epidemic. "Infected day" is when we identified the infection. The range of consecutive days, including the infected day, is "Period Y." In defining the Period Y, *n* shows the number of days before the “infected days”; *m* shows the number of days following. Thus, the number of days in Period Y is *n* + 1 + *m*. For example, if *n* = 2 and *m* = 3, the calculation is Y = 2 + 1 + 3 = 6. In Fig 4, X + Y + Z represents the entire length of the infection period: Period X is the length of the "infected day" minus *n*; Period Z is the length of the “End of the epidemic” minus X + Y.

*2*.*3*.*2*.*2 [Definition] (discrimination failure rate)*. Y is the number of farms estimated infected around the time of infection (Fig 4-Y). X is the number of farms never infected or predicted affected earlier than the infected time (Fig 4-X). Z is the number of farms where we overlooked the virus or infected after the infected time (Fig 4-Z). We determine the failure rate FR as follows, with the false positive rate FP, the false negative rate FN, the sensitivity SE, and the specificity SP.

FR=FP+FN (5)

FN=1–SE,SE=(x+y)/(x+y+z) (6)

FP=1–SP,SP=(y+z)/(x+y+z) (7)

As described above, a false negative occurs when we misjudge the infected condition as negativity throughout the end of infection (period Y). A false positive occurs when we judge the status as a positive one or more times before the infection day (Fig 4-X). The discrimination failure rate is the ratio of false negatives and false positives among all the judgment target farms.

We constructed a performance evaluation graph adopting the proposed method with the Miyazaki data (2.1). As a result, we could produce a valley-shaped graph with a singular minimum value (Fig 3-(1)). The shape indicated that the discriminant ability of the FMD-VS was valid. The discriminating ability becomes maximum depending on the view of the vertex when we set the threshold to 30 (*λ* = 0.006).

Fig 3-(1) shows the values of *n* and *m* defining the vicinity of the infection date were *n* = 5 and *m* = 0, which mean Y = 5 + 1 + 0 = 6 correspond to an “around the time of infection” setting of 6 days. As described in Section 3, changing parameters *n* and *m* change the kurtosis of the graph. We performed verification experiments by setting multiple values between 1≦Y≦10 for parameters *n* and *m*. Under this condition, we confirmed that the larger Y is, the greater the kurtosis becomes, and the peak tends to become sharper.

#### 2.3.3 Improvement of accuracy

In Section 2.3.1, we pointed out the possibility that the histogram (Fig 1) created from the Miyazaki Prefecture data may not represent the true distribution needed for our analysis. We focused on the small number of data and the uncertainty of the estimated infection date as the causes. As another cause, we focused on the fact that the conditions of each farm are different and devised a method of improving the accuracy of FMD-VS based on this point. [34]. Stratification classifies the entire sample according to a particular factor. If we perform an appropriate stratification, it will divide the entire sample into homogeneous groups. Then, the histogram created for the various groups will provide a better representation of the true distribution than the one before stratification. We considered that such stratification could improve the accuracy of FMD-VS. However, we were uncertain of the factors that would be more effective for improving accuracy. For this reason, we first tried to confirm the effective factors by improving accuracy. First, we listed candidates for stratification factors such as animal species, farm size, time of infection, and geographical conditions. For each factor, we followed the next two-stage test.

Stage 1. Stratify the histogram of the entire sample based on one aspect of interest. Compare the individual histogram after stratification to the shape of the original histogram and test the stratification effect. We judge the factor if we decrease the irregularity of the resulted histogram compared to the original histogram with a shape closer to the desired bell-shape. The set of factors passing this initial screening would then undergo a stricter evaluation, as described in Stage 2.

Stage 2. To confirm whether the performance evaluation graph based on the histogram, generated after stratification by each of the selected factors, shows better accuracy than the original histogram without stratification. Compare the minimum value of the graph and judge the factor effective if the minimum rate after stratification is smaller than the initial minimum value.

If the factor of interest is the scale of the farm, then we could classify the farms into two types, large and small. We would then create models for performance evaluation (see 2.3.2, Fig 3-(2)), cumulative relative frequency distributions, for each farm type (*M1* and *M2*). However, since the performance evaluation should be a comprehensive evaluation of the entire sample irrespective of stratification, a single performance evaluation would need to be combined with *M1* and *M2*. Threshold *I*_*T*_ (0 ≤ *I*_*T*_ ≤ 100) determines the measurement accuracy *a (I*_*T*_*)*. Discriminate the ability check described in 2.3.2 for *M1* and *M2*. Observe each of the discrimination failure rates and calculate *a(I*_*T*_*)* as the mean of the two failure rates.

The model determines the scattering index associated with the threshold *I*_*T*_. Therefore, for this example, the corresponding scattering indices exist for the two models *M1* and *M2*, such as *λ*_*IT*_^*M1*^ and *λ*_*IT*_^*M2*^.

Experimental results (refer to Section 3) show that large farms have smaller values (*λ*_*IT*_^*M1*^ <*λ*_*IT*_^*M2*^), indicating that large farms are contagious with a poor scattering index, it infects them at a stage with less scattering. In this way, it is the element of the accuracy improvement method that we propose that is criteria for determining the change to infectivity can be set separately for each farm characteristic.

#### 2.3.4 Procedure for generating a performance evaluation graph

The following is the detailed procedure used to generate a performance evaluation graph considering the stratification effect: We identify the day we confirmed the infection in the data and seek the scattering index from the first to the last of the infection period for all farms listed in the Infection Data (2.1). In short, create a chronological order of dispersion index for each farm. We prepare a variable “Number of discrimination failures” *S*_*IT*_ (0 ≤ *I*_*T*_ ≤ 100) for calculating the discrimination failure rate, reset all (*S*_*0*_ = 0, *S*_*1*_ = 0, …, *S*_*100*_ = 0) and set the threshold *I*_*T*_ to 0 (*I*_*T*_ = 0).

1. Stratify all target farms according to the factors of interest. (Number of stratification is *n*_*1*_) We set the number of cross-validation as *n*_*2*_ (*n*_*2*_ = 100 for this time) and set the variable *j* (0 ≤ *j* ≤ *n*_*2*_) for counting the number to 0.
2. Select one of the farm sets achieved by stratification.
3. Create a model (2.3.2) for performance evaluation for a part of all target farms and use the rest of the farm data for test data. Increase the number of cross-validations from *j* to 0.
4. Normalize the scattering index of the horizontal axis of the model from 0 to 100.
5. Select one of the test data-target farms. (The number of test data is n3.)
6. From the chronological order of the scattering index of the farm, if it exceeds the scattering index associated with *I*_*T*_ for the first time around the time of infection of the farm, we judge that the discrimination under *I*_*T*_ is a success, otherwise; it is a failure. In case of failure, increase the number *S*_*IT*_ of discrimination failures by 1.
7. If the test data selection is complete, go to 9; otherwise, go back to 6 and select another test data (farm).
8. If *I*_*T*_ = 100, go to 10. If (*I*_*T*_ <100), set *I*_*T*_ + 1 to the current *I*_*T*_, set this *I*_*T*_ + 1 as a new *I*_*T*_, and return to 6.
9. Go to 11 if the number of cross-validations in 4 has reached *n*_*2*_. If not, go back to 4 and replace the data for model creation with the test data.
10. Go to 13 if the selection of farm set obtained by stratification in 3 is completed. If not, go back to 3 and select another farm set.
11. Plot each point (*I*_*T*_, *a(I*_*T*_*)*) (0 ≦ *I*_*T*_ ≦ 100) in the performance evaluation graph. The discrimination failure rate *a(I*_*T*_*)* is as follows.

$$a\left(I_{T}\right)=S_{IT}/n_{1}n_{2}n_{3}$$ (8)

The product of the stratified number *n*_*1*_, the number of cross-validation *n*_*2*_, and the number of test data n3 is the total number of trials for each threshold *I*_*T*_. *m* in formula (4) is synonymous with *n*_*2*_ *n_*3*_.

#### 2.3.5 Example of stratification and creating performance evaluation graph

As a result of the histogram-based analysis (2.3.3–1) applying the infection data (2.1) of the Miyazaki Prefecture, we judge that the following three types were likely to improve the performance of FMD-VS: animal species, farm size, and distance from the center of gravity of the entire infected farm (abbreviated as the distance from the center of gravity). Since the stratification method regarding animal species and farm-scale is clear, we show below the stratification focusing on the “distance from the center of gravity” and the performance evaluation graph based on this.

**Fig 5:**
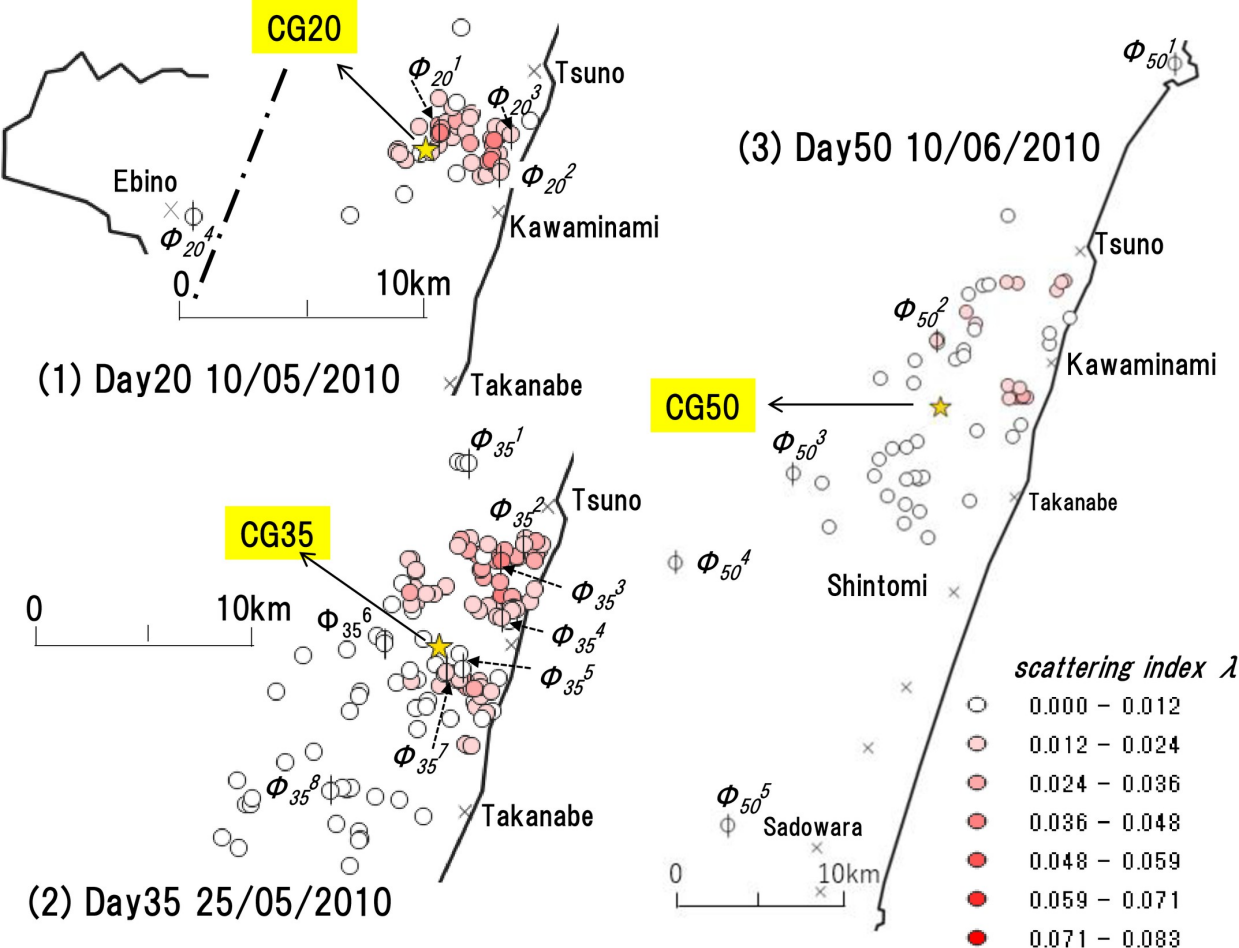
Distance from the center of gravity of the entire infected farm. (1), (2), (3) represents the infection situation on the 20th (10/05/2010), 35th (25/05/2010), 50th (10/06/2010) day after the outbreak. In each case, the circles show the infected farms. Circles with vertical lines show the farms confirmed infected on that day. The color gradation of each farm (circle) represents the indensity of the scattering index *λ* of the day of interest. Asterisks (CG20, CG35, and CG50) show the center of gravity (referred to as the "center of gravity") of the entire infected farm (circle) on each day. For example, the circle in (1) represents the farm infected 20 days after the outbreak, and the asterisk CG20 represents the "center of gravity" of the entire farm. The farms confirmed infected on this day (Day 20) are the four circles with vertical lines, and the indexes Φ_20_^1^, Φ_20_^2^, Φ_20_^3^, and Φ_20_^4^, respectively.

*2*.*3*.*5*.*1 Stratification focusing on "distance from the center of gravity"*. First, we will explain the “entire infective farm” and “center of gravity” using Fig 5. The map (1), (2), (3) in Fig 5 shows the infection status extracted from the infection period shown in the official infection data for 2010. The infectious period is from April 20^th^, 2010, to July 5^th^, 2010 (denoted as Day0-Day65). If April 20^th^, 2010, is day 0 (Day 0), (1) corresponds to the 20th day (Day20: May 20^th^, 2010), (2) corresponds to the 35th day (Day35: May 25^th^, 2010), and (3) corresponds to the 50th day (Day50: June 10^th^, 2010). The circles, including circles with a vertical bar marked as index *Φ*, on each map represent the farms infected on the day of focus. It shows the farms identified infected by the day of focus, and where slaughter and burial not executed by that day. The “entire infected farm” stands for the entire section of circles, and the center of gravity of it is the “center of gravity” (referred to as “center of gravity”). In the figure, an asterisk represents the center of gravity and is referred to as CG. If the day of interest is the center of gravity of Day k (k = 0, …, 65), we label it CGk. A circle with a vertical line shows the area of the farms where we identified the infection on the day of interest, referred to as “*Φ* farm.” We marked the *Φ* farm on day k (that is, Day k) after the outbreak as *Φ*_*k*_ farm. We add an index *j* for distinguishing each farm and note as *Φ*_*k*_^*j*^. The tint of the circles shows the magnitude of the value of *λ* for each farm.

Based on the above, for example, the circles show the areas of the farms infected on Day 20: May 10^th^, 2010. Among them, four farms were infected on this day (Day 20), *Φ*_*20*_, which area is represented by *Φ*_*20*_^*1*^, *Φ*_*20*_^*2*^, *Φ*_*20*_^*3*^, and *Φ*_*20*_^*4*^. The asterisk (CG20) shows the center of gravity of all the circles, and color gradation shows the *λ* value of each farm on this day. In the same way, in Fig 5, the circles show the farms infected on Day35: May 25^th^, 2010, which is eight *Φ*_*35*_ farms. *Φ*_*35*_^*1*^ to *Φ*_*35*_^*8*^ represents each area.

Day50: June 10^th^, 2010, is still the same, there are four *Φ*_*50*_ farms, and *Φ*_*50*_^*1*^ to *Φ*_*50*_^*4*^ represents the area of each.

**Fig 6:**
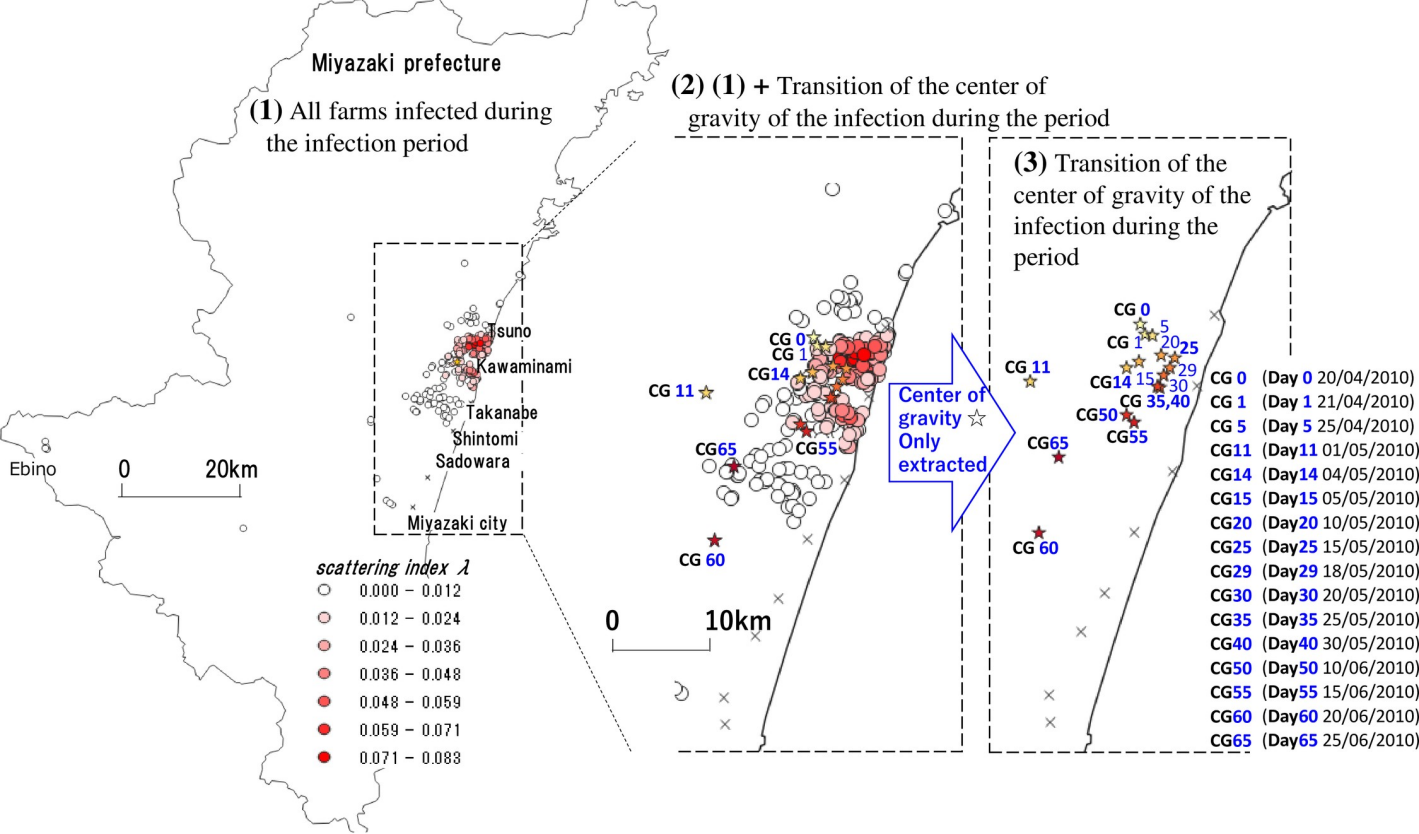
Location of all infected farms and the transition of the center of gravity during the infection period. In Fig 6- (1), the circles show the area of all farms infected during the infection period (Day 0 (20/04/2010) to Day 65 (25/06/2010)). The tonal gradation shows the scattering index of “the date of confirmed infection” of each farm. Fig 6-(2) plots some centers of gravity (CG0, CG1, CD5, …, CG65) that shift during the infection period in Fig 6-(1). Fig 6-(3) shows only the center of gravity (CG0, CG1, CD5, …, CG65) of Fig 6-(2).

As a supplementary explanation, Fig 6 shows the transition of all 292 infected farms and the center-of-gravity CGk (k = 0,…, 65) during the infection period. The viewpoint of the figure is that the circles in Fig 6-(1) represent all the areas of infected farms. The shading shows the size of the value *λ* for the infection identified on each farm. In Fig 6-(2), we extract and plot some centers of gravity which change during the infection period with asterisks (CG0, CG1, CG5, …, CG65), together with the distribution of the circles in Fig 6- (1). Fig 6-(3) shows only the transition of the center of gravity.

**Fig 7:**
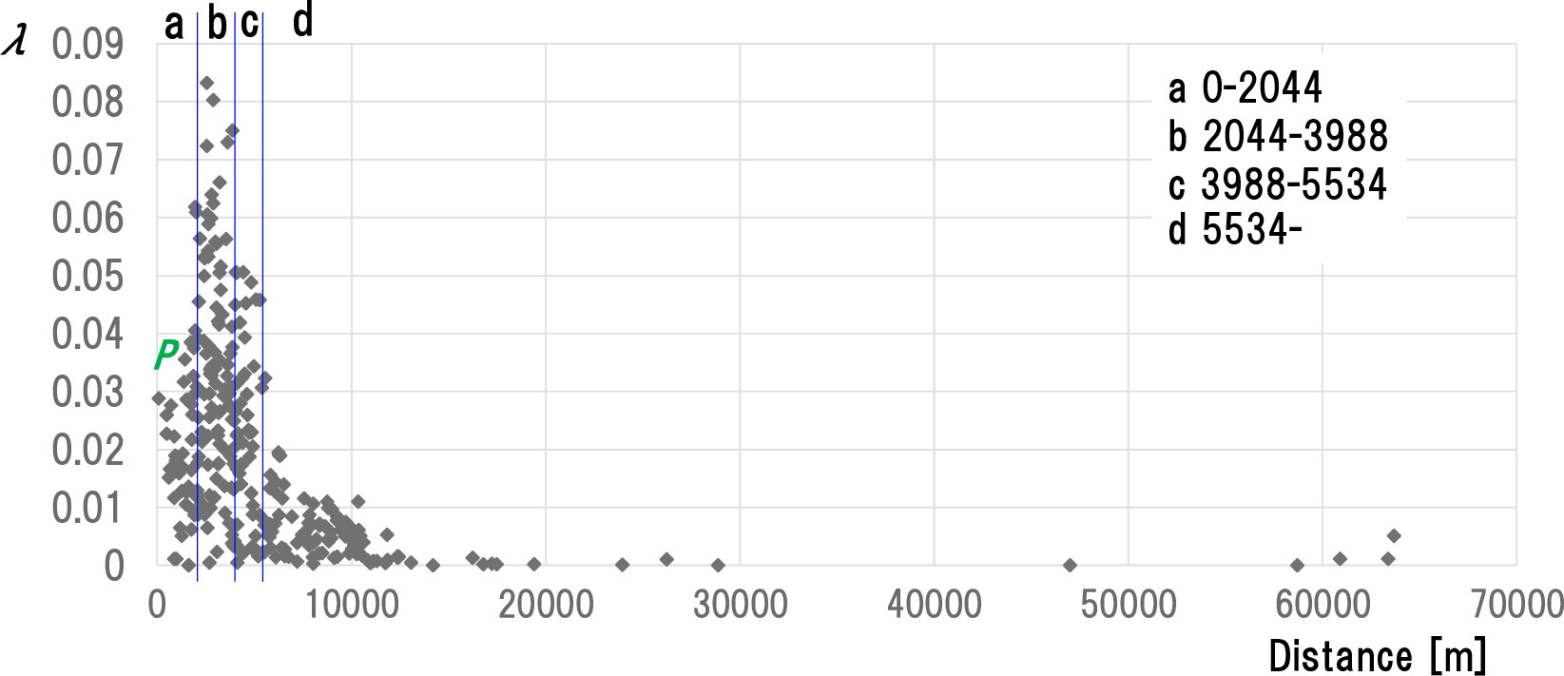
Stratified results based on the distance from the center of gravity. The horizontal axis represents the distance from the center of gravity (CG0 or CG1 or CD2 or… or CG65). The vertical axis represents the scattering index λ. It associates each point with the 292 farms infected during the infection period. For example, point *P* (1586, 0.0365) associates with one farm (Φ_20_^1^) confirmed infected on the 20th day (Day 20) after the outbreak. 1586 represents the distance from the center of gravity (CG20) of the day (Day 20) for the farm (Φ_20_^1^), and 0.0365 represents the scattering index for the farm (Φ_20_^1^) on Day 20. We performed stratification based on the distance from the center of gravity for all points in the above graph. The cluster area obtained from the stratification result is a, b, c, d. Here, region a is 0 or more and less than 2044m from the center of gravity (CG0 or CG1 or CD2 or … or CG65). b is 2044m or more and less than 3988m, c is 3988m or more and less than 5534m, and d is 5534mor more.

Based on the above, we will explain the stratification of the “distance from the center of gravity.” Fig 7 shows the results.

The horizontal axis represents the distance from the center of gravity (CG0, CG1, CD2 to CG65). The vertical axis represents the scattering index *λ*. We associate each point on the graph with each of the 292 farms infected during the infection period. For example, point *P* (1586, 0.0365) associates with one farm (“*Φ*_*20*_^*1*^”) found infected 20 days after the outbreak (Day 20). “1586 (m)” represents the distance from the center of gravity (CG20) of the day (Day 20) to the farm (*Φ*_*20*_^*1*^). “0.0365” represents the dispersion index of the farm (*Φ*_*20*_^*1*^) on Day 20.

All the dots in the above graph are sample data for stratification. The obtained cluster areas are a, b, c, d. Area a shows that the distance from the center of gravity (CG0, CG1, CD2 to CG65) is 0m or more and less than 2044 m. b is 2044 m or more and less than 3988, c is 3988 m or more and less than 5534 m, and d is 5534 m or more.

We will explain (I) the sample data for the stratification and (II) its method in more detail below.

### (Ⅰ) The sample data for stratification (1)

The horizontal axis of the graph represents the center of gravity on the ordinal date k (k = 0,…,65) after the outbreak, the distance (*d*_*kj*_)) from CGk to the farm *Φ*_*k*_^*j*^ (j is an index for distinguishing individual farms) where we identified the infection.

We express the distance (*d*_*kj*_) as below.

$$\sqrt{{\left(x_{p}-\frac{1}{m}\sum_{\left(i=1\right)}^{m}x_{i}\right)}^{2}+{\left(y_{p}-\frac{1}{m}\sum_{\left(j=1\right)}^{m}y_{i}\right)}^{2}}$$ (9)

(*x*_*i*_, *y*_*i*_) is the position coordinates of farms infected on Day *k*, m is the total number, and (*x*_*p*_, *y*_*p*_) is the position coordinates of the farms. The vertical axis represents the dispersion index (*λ*_*kj*_) on Day k of farm *Φ*_*k*_^*j*^ identified infected on Day k.

**Fig 8:**
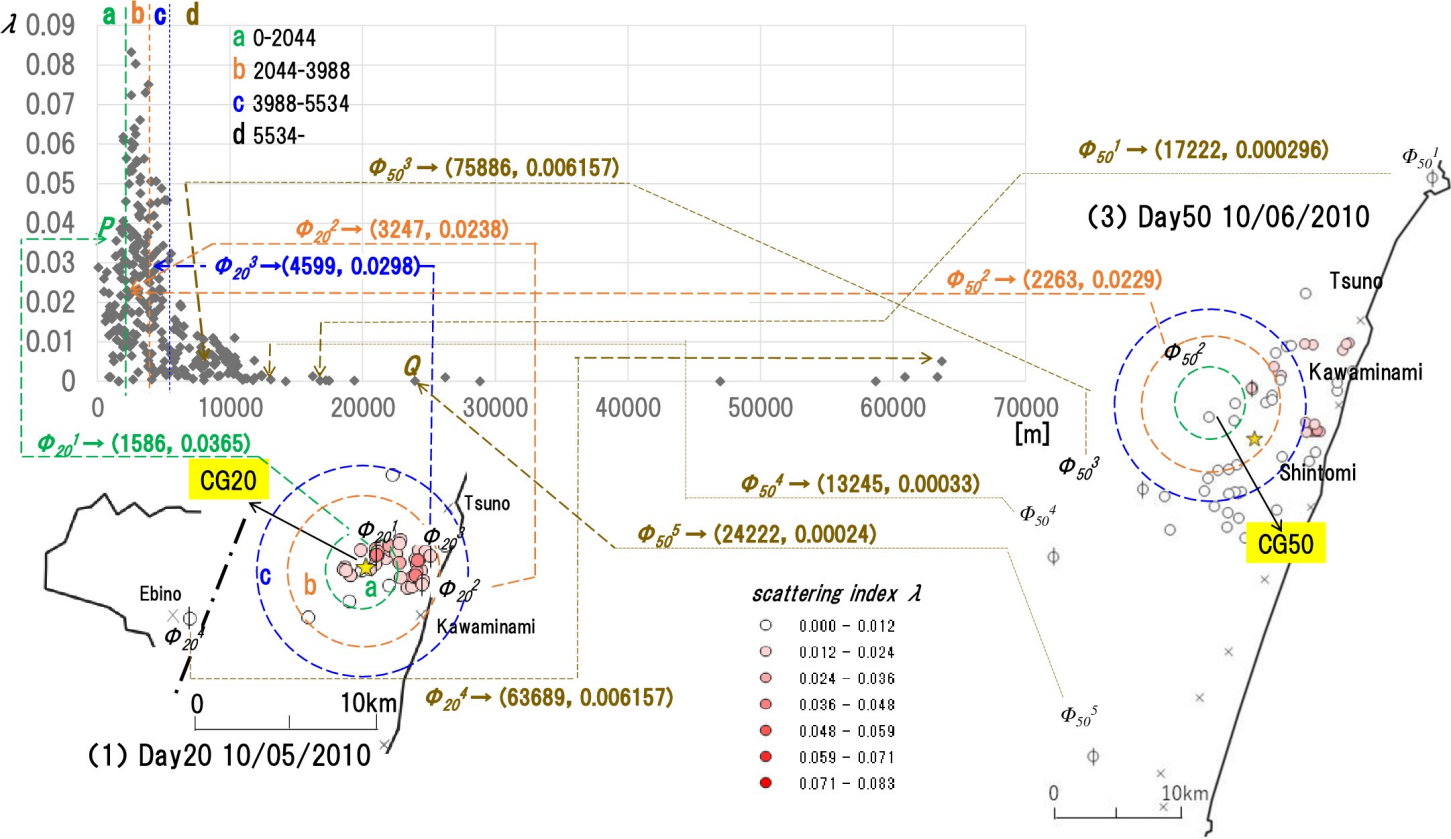
Definition of points that construct the graph in Fig 7. We explain points P and Q with maps (1) and (3) in Fig 5. The radius of the concentric circle a is 2044 m, which is the uppermost limit of the cluster area a. That of b is 3988m, and that of c is 5534m. The boundaries of a and b, b and c, and c and d in Fig 7 are semantically equivalent to the contours of concentric circles a, b, and c. First, we plot point P according to the farm labeled Φ_20_^1^ on map (1), infected on Day 20(10/05/2010). 1586m of the coordinates (1586, 0.0365) for point P represents the distance from the center of gravity (CG20) to farm Φ_20_^1^ on this day (Day 20), and 0.0365 represents the scattering index. We plot point Q according to the farmlabeled Φ_50_^5^ on map (3), infected on Day 50(10/06/2010). 24222m of the coordinates (24222, 0.00024) for point Q represents the distance from the center of gravity (CG50) to farm Φ_50_^5^ on this day (Day 50), and 0.0024 represents the scattering index. We figure and plot the others in the same method as of P and Q above. The other points can be defined in the same way as P and Q described above.

In summary, each point in the graph of Fig 7 represents the distance *dkj* from the center-of-gravity CGk of the farm *Φ*_*k*_^*j*^ infected on the ordinal date k, and by the ordered pair (*d*_*kj*_, *λ*_*kj*_) of the scattering index *λ*_*kj*_. “*Φ*_*k*_^*j*^” and “(*d*_*kj*_, *λ*_*kj*_)” are synonymous. Using Fig 8, we will show how we established the above sample data. Maps (1) and (3) in Fig 8 are the same as (1) and (3) in Fig 5. The graph in Fig 8 is the same as the graph in Fig 7. Area a, b, c separated by concentric circles on the map in Fig 8 associates with cluster areas a, b, c separated by vertical lines. We will describe the stratification method later on (II). We have plotted the dots in the graph in Fig 8 (i.e. graph in Fig 7) by associating them with each farm *Φ*_*k*_ (k = 0,…, 65) that appears during Day 0 to Day 65. Fig 8 shows this with *Φ*_*20*_ and *Φ*_*50*_ farms. First, for farm *Φ*_*20*_, for example, dot P (1586, 0.0365) at the tip of the arrow with *Φ*_*20*_^*1*^ (1586, 0.0365) associates with farm *Φ*_*20*_^*1*^ on map (1).

*Φ*_*20*_^*1*^ (1586, 0.0365) indicates that farm *Φ*_*20*_^*1*^ was infected on the 20th day (Day 20) after the outbreak. Distance from the center-of-gravity CG20 is 1568m, with a dispersion index of 0.0365. A Dot (3247, 0.0238) at the tip of the arrow marked *Φ*_*20*_^*2*^ (3247, 0.0238) associates with farm *Φ*_*20*_^*2*^ on map (1). *Φ*_*20*_^*2*^ (3247, 0.0238) shows that farm *Φ*_*20*_^*2*^ was infected on the 20th day (Day 20) after the outbreak. Distance from the center-of-gravity CG20 is 3247m, with a dispersion index of 0.0238. Dot (17222, 0.000296) at the tip of the arrow marked *Φ*_*50*_^*1*^ (17222, 0.000296) associates with farm *Φ*_*50*_^*1*^ on map (3). *Φ*_*50*_^*1*^ (17222, 0.000296) shows that farm *Φ*_*50*_^*1*^ was infected on the 50th day (Day 50) after the outbreak. Distance from the center-of-gravity CG50 is 17222m, with a dispersion index of 0.000296. Dot (2263, 0.0229) at the tip of the arrow marked *Φ*_*50*_^*2*^ (2263, 0.0229) associates with farm *Φ*_*50*_^*2*^ on the map (3). *Φ*_*50*_^*2*^ (2263, 0.0229) shows that farm *Φ*_*50*_^*2*^ was infected on the 50th day (Day 50) after the outbreak. Distance from the center-of-gravity CG50 is 2263m, with a dispersion index of 0.0229.

### (Ⅱ) Stratification method

We will explain the stratification method of the above sample data (cluster of dots in the graph of Fig 7 or 8).

Here, we used Shapiro-Wilk's normality test method [35] as a method for stratifying each data group for easy statistical estimation.

First, we determine whether the clusters of *λ* up to the ordinal N (N = 20 in this case) meet the normality in ascending order of distance from the center-of-gravity CG k (k = 0, …, 65). If there is fulfillment, we add the ordinal N+1 *λ* to the cluster and repeat the judgment performance.

As described above, we repeat the procedure until the normality of the cluster no longer fulfills at ordinal N+*α*. Then, we set the previous set of data (N + *α*-1) as a cluster region (here as a). Next, we start with the perception of the normality of the ordinal (N + *α*) data to the ordinal (N + *α* + 19) cluster and follow the same procedure to find the region b. The same applies to region c. Region d consists of all the remaining data excluded in regions a, b, and c without conducting judgment of normality. We determined the number of clusters regions to be four this time.

The cluster regions obtained by the above procedure are the four regions a, b, c, and d in Fig 7. The cluster area closest to the center-of-gravity GCk (k = 0, ‥, 65) is a, next is b, next c, and the farthest is d. The data number in clusters a, b, c, d are 42, 96, 54, and 100. Table 1 shows the mean and standard deviation of *λ* in each cluster regions. The mean and standard deviation of *λ* of clusters a and c in Table 1 are almost the same. Those of b, between a and c, are greater than a and c.

**Table 1:** Quantitative analysis of cluster regions obtained by stratification by distance from “Center of Gravity".

	Sizes of the clusters (m)	The number of data	Average of *λ*	Dispersion of *λ*
**a**	**0–2044**	**42**	**0.020962**	**0.014108**
**b**	**2044–3988**	**96**	**0.032653**	**0.019347**
**c**	**3988–5534**	**54**	**0.021084**	**0.015382**
**d**	**5534-**	**100**	**0.005352**	**0.005231**

Table 1 shows the mean and standard deviation of *λ* for each cluster region. This table expresses that the mean and standard deviation of *λ* for clusters a and c are almost the same. The mean and standard deviation of b between a and c are higher than those of a and c.

From the visual image of the graph in Fig 7, the value and variation of *λ* of cluster a increase as it gets nearer to b, and that of c decrease as it gets farther from b.

**Fig 9:**
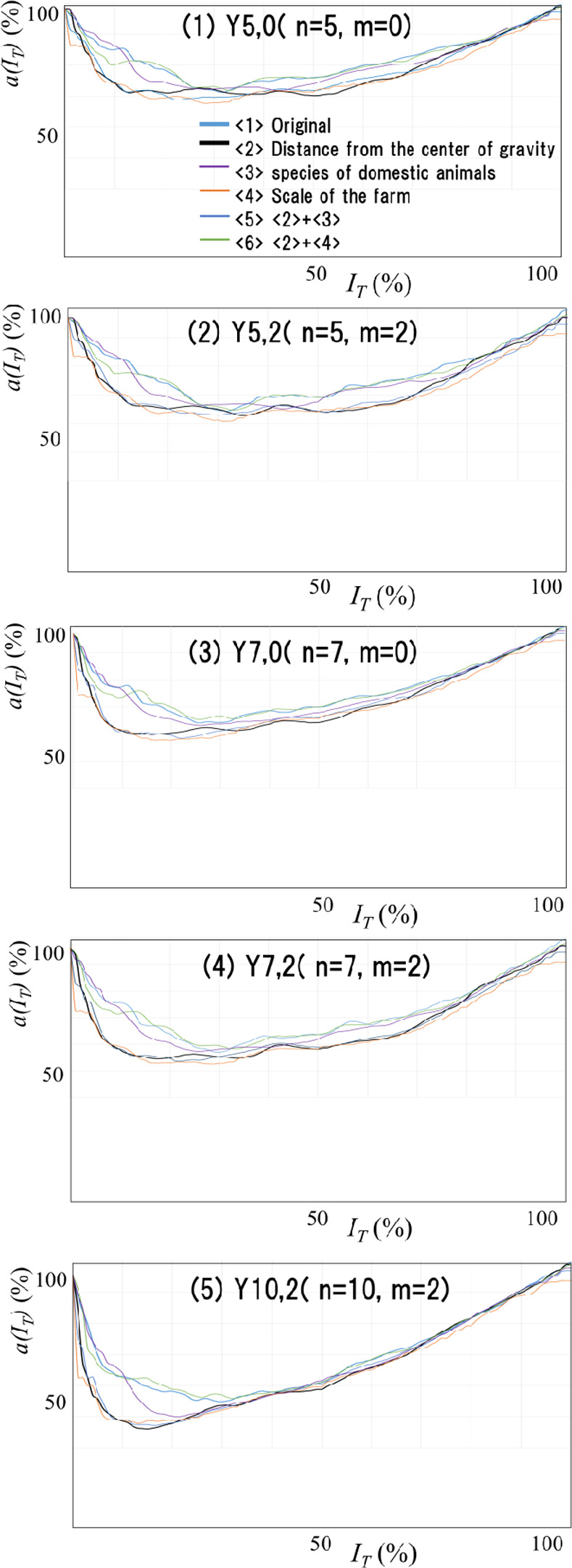
Performance evaluation graph for various FMD-VS. Fig 9 shows a part of the performance evaluation graph that is the basis of the evaluation results. The performance evaluation graphs of FMD-VS of 5 (patterns) x 6 (species) = 30 variations (by all the combination of no stratification (<1>) and five types of strata (<2> to <6>) and settings of 5 out of 8 patterns around infection day Y5,0, Y5,2, Y7,0, Y7,2, Y10,2) are created and shown in (1) to (5). The least value in each graph is the performance of each FMD-VS. The graph visually shows that the performance of FMV-DS constructed by stratification based on the distance from the center of gravity is high, and the performance is higher as the width of the period set around infection day is larger.

We applied the performance evaluation generation procedure (2.3.4) to the FMD-VS stratified by the above results. <2> in Fig 9-(1) to Fig 9-(5) are the results described in bold black lines. The examination and interpretation are the same as in Fig 3-(1). We will explain the differences of (1) to (5) in Fig 9 and about the graphs other than <2> in the next section.

## 3. Results and discussion

### 3.1 Evaluation experiment

First, we constructed six models of FMD-VS with five characters of stratification besides the original pattern (<1>) without stratification. As mentioned at the beginning of section 2.3.5, three of the five characters are a distance from the center of gravity (<2>), animal species (<3>), and farm size (<4>). The remaining two types are a mixture of <2> and <3>, and a combination of <2> and <4> (<2>+ <3>, <2> +<4>). We classified the cluster area (<2>) into four as described in 2.3.5- (II), animal species (<3>) into two areas of cattle and pigs and the farm size (<4>) into three areas: large (1000 or more), medium (100 to 999), and small (99 or less).

In <2> + <3>, we set eight areas for all combinations of four in <2> and two in <3>. And for <2>+<4>, twelve areas for all combinations of four of <2> and three of <4>.

On constructing the FMD-VS, we examined the eight patterns. We marked “Y_n,m_” as around the time of infection, which is (n+1+m) days, from n days before and m days after the infection identified according to the infection data. Y_n,m_ is defined by 8 patterns: Y5,0, Y5,1, Y5,2, Y7,0, Y7,1, Y7,2, Y10,0, Y10,2. Here we set the shortest setting to Y5,0, which is n +1 m = 6 (n = 5, m = 0). The reason for setting a period of 6 days is the minimum number of days required for the shape of the performance evaluation graph to be *an irregular valley-shape with one vertex*, which suggests statistical significance. We have accidentally found this under this precondition. We set n as the breakdown of n and m as large as possible before the infection day is because it is relevant to suppress the false-negative rate that increases the feasibility of the outbreak.

We set the longest period “around the time of infection” as n + 1 + m = 13 days (n = 10, m = 2) based on the following concept. The kurtosis becomes sharper as the period of Y_n,m_ near the infected day is increased to six days, seven days, and so on. However, the larger the period of Y_n,m_, the larger the ratio that allows the deviation from the initial data. Thus, we set the greatest permissible difference as the longest period of Y_n,m_ to 13 days (n = 10, m = 2).

The reason we set n = 10 is that the incubation period for FMD was 6.2 days for cattle and 10.6 days for pigs that we judged appropriate. Also, m = 2 is a given value because infection confirmation was not performed daily. This is the basis for the shortest of Y5,0 and the longest of Y10,2 in the period set for Y_n,m_ around the time of infection. The intermediate values Y5,1, Y5,2, Y7,0, Y7,1, Y7,2 are the periods set with appropriate intervals within the range of 5 ≦ n ≦ 10 and 0 ≦ m ≦ 2. Based on the above, we conducted evaluation experiments with 6 (characters) x 8 (patterns) = 48 variations of performance evaluation graphs of FMD-VS.

### 3.2 Results and discussion

Table 2 shows the results of the evaluation experiment. There are 48 variations of FMD-VS by combinations of 6 characters of stratification and eight patterns around the time of infection described in Section 3.1. Each row represents six characters (<1>, <2>, <3>, <4>, <2> + <3>, <2> + <4>) for each stratification and each column represents 8 patterns (Y5,0, Y5,1, Y5,2, Y7,0, Y7,1, Y7,2, Y10,0, Y10,2) of “around the time of infection”. Each numerical value in the table represents the performance of the corresponding FMD-VS. However, the minimum value of the performance evaluation graph represents this performance. Therefore, the smaller the value, the greater the performance is.

**Table 2:** Results of evaluation experiment (performance comparison for FMD-VS).

		5a	5b	5c	7a	7b	7c	10a	10c
		Y5,0	Y5,1	Y5,2	Y7,0	Y7,1	Y7,2	Y10,0	Y10,2
		n = 5, m = 0	n = 5, m = 1	n = 5, m = 2	n = 7, m = 0	n = 7, m = 1	n = 7, m = 2	n = 10, m = 0	n = 10, m = 2
**<1> no stratification**		**71.20**	**67.43**	**64.30**	**64.19**	**60.07**	**57.05**	**54.95**	**46.73**
**<2>-<6> stratification**									
**<2>**	**Distance from the center of gravity**	**70.17**	**67.46**	**62.88**	**59.89**	**57.43**	**54.53**	**46.04**	**41.19**
**<3>**	**species of domestic animals**	**72,7**	**68.05**	**64.65**	**65.44**	**61.35**	**58.34**	**56.66**	**48.82**
**<4>**	**Scale of the farm**	**71.86**	**67.13**	**64.87**	**63.23**	**60.79**	**57.54**	**50.05**	**49.19**
**<5>**	**<2>+<3>**	**67.67**	**63.56**	**60.79**	**57.76**	**54.12**	**52.91**	**47.28**	**48.15**
**<6>**	**<2>+<4>**	**68.89**	**65.24**	**62.99**	**58.49**	**55.87**	**53.58**	**48.04**	**42.63**
									**(%)**

We made 6 × 8 = 48 varieties of FMD-VS, with combinations of no stratification (<1>) and five types of strata (<2> to <6>), and eight patterns of infection date settings (Y5,0, Y5,1, …, Y10,0, Y10,2), and created a performance evaluation chart each. We then tested the performance of each FMD-VS with the least value in the diagram. Since the value in the graph is the discrimination failure, the smaller the value, the bigger the performance a corresponding FMD-VS can show. From this table, we can see that the performance of FMV-DS constructed by stratification based on the distance from the center of gravity is higher and that the performance is higher as the width of period set around infection day is larger.

Fig 9 shows a part of the performance evaluation graph that is the basis of the results in Table 2. We extract five patterns: Y5,0, Y5,2, Y7,0, Y7,2, Y10,2, out of 8 patterns of Y_n,m_ around the time of infection. We combine each with six characters of strata: <1> no stratification, <2>distance from the center of gravity, <3> animal species, <4> farm size, <5> <2>+<3>, <6> +<2>. Fig 9- (1) to Fig 9-(5) show the performance evaluation graphs of FMD-VS with 5 (patterns) x 6 (characters) = 30 variations. The six graphs in Fig 9-(1) represent the FMD-VS performance evaluation graphs constructed for each of the six stratifications under the setting of Y5,0 around the time of infection. The same applies to Fig 9-(2) to Fig 9-(5).

From Table 2 and Fig 9, which shows the results of the above evaluation experiments, we could perceive the following. First, the discrimination failure rate in cases (<2>, <5> <2> + <3>, <6> <2> + <4>) regarding the distance from the center of gravity of the entire infected farm is low. FMV-DS constructed by stratification based on the distance from the center of gravity is high-performance. We again found that FMV-DS performance improved as the width of the period set around the time of infection increased. However, setting this period to a substantial value also increases the ratio that allows the deviation from the actual data. For this reason, it is necessary to have rational reasons for determining the maximum for this set period. The basis for this period setting is as described above.

## 4. Performance of FMD-VS and utilization at the outbreak materials and methods

To this point, we have described the system of our proposed FMD-VS, along with its analysis and evaluation performance. We have also shown ways of improving its accuracy. We will observe future outbreaks of infection and describe the tools to be provided to government officials, including guidelines for their usage.

### 4.1 Performance of FMD-VS

From the experimental results of the previous section, we found that information about the distance from the center of gravity of the entire infected farm contributes to the performance improvement of FMD-VS. Stratification by animal species had little impact. The following are the reasons for this:

- Information on animal species is already incorporated into *λ* in the Keeling model as an inter-farm transmission rate. Thus, stratification by animal species does not provide additional information.
- The Keeling model does not include information regarding the barycentric position of the infected farm. Thus, we assume stratification adds that extra information based on the center of gravity.

### 4.2 Utilization at the time of the outbreak

Our principal aim was to develop a system that supports the judgment of government officials on epidemic prevention (referred to as a support system) easier to understand and operate without modeling expertise. Thus, we recognized the need to show how and where to use FMD-VS. Preventing epidemics includes various restrictions such as movement restrictions on people, cars, livestock, and setting disinfection sites. To clarify the performance of FMD-VS, we will focus on the “selection of farms for culling,” where the influence of epidemic judgment has a high impact. The proposed support system pursues the following structure: First, (1) narrow the range of thresholds for epidemic judgment. The narrowing for (1) is performed logically to some extent; FMD-VS and the performance evaluation graph are used as tools for this purpose. Then in step (2), based on the results of step (1), probability produces the threshold selection (decision-making). The idea is to identify the constraints: budgets, personnel, supplies, burial sites, relevant to the infected area and to set the best threshold consistent with constraints. We describe the method for (1), which narrows the range of threshold possibilities. We will report on (2), making the threshold decision after identifying the relevant constraints, which will be in another article, as this is under development.

#### 4.2.1 Threshold for prevention of epidemics judgment

**Fig 10:**
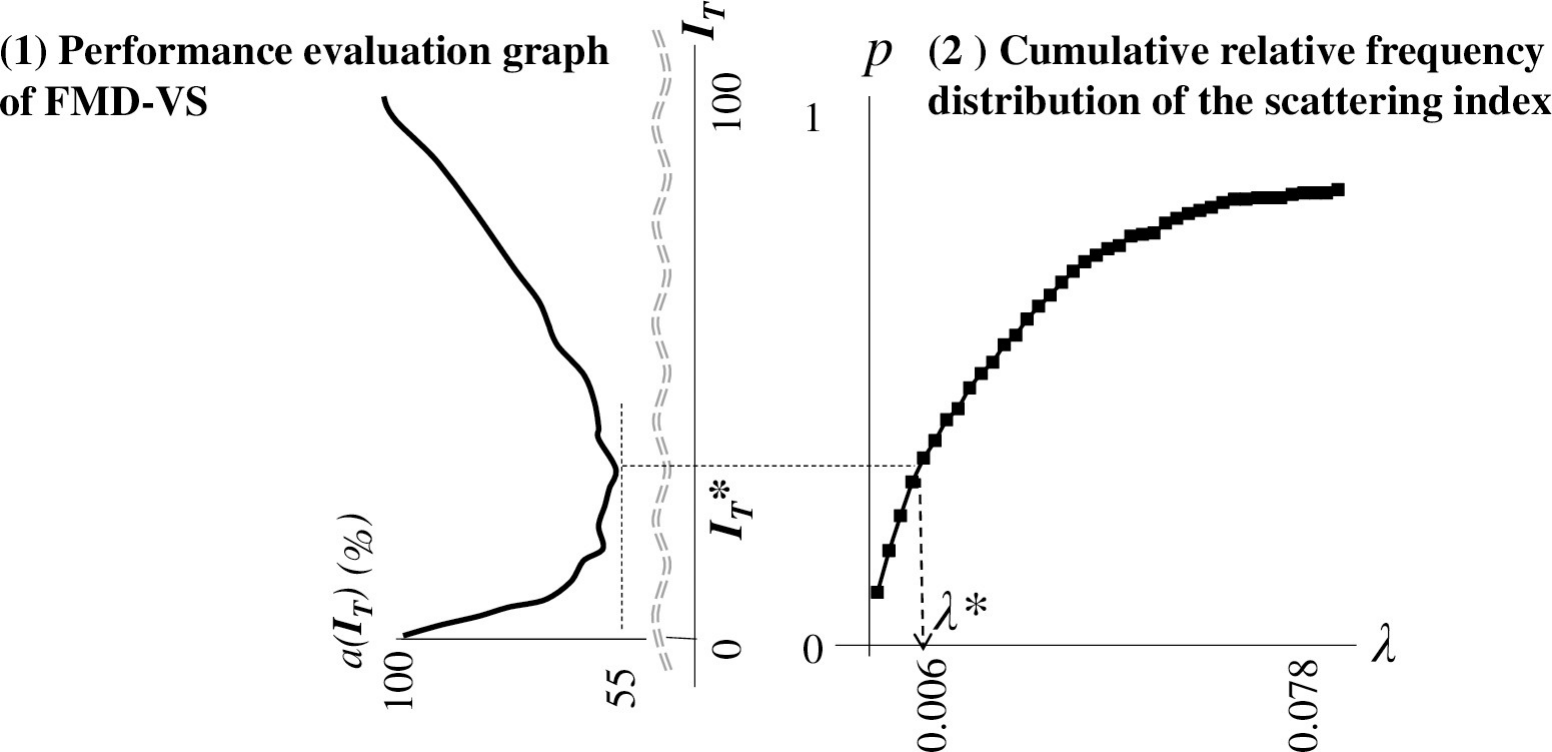
Application example of FMD-VS for prevention of epidemics judgment. When the associated scattering index λ * is 0.006 for the threshold IT * selected for prevention of epidemics, farms with a scattering index exceeding 0.006 are considered to be destroyed at the point.

We use the FMD-VS performance evaluation graph (Section 2) to narrow the threshold values for epidemic judgment. Fig 10-(1) shows the chart and Fig 10-(2) illustrates “the cumulative frequency distribution of the scattering index,” the actual state of the FMD-VS, the object of the evaluation (refer to Fig 3). For example, suppose λ* is the scattering index selected as the threshold for epidemic judgment, and λ* is associated with IT*. If λ* is 0.006, then it destroys the animals on the farms with a scattering index above 0.006.

What would be the best threshold IT* for epidemic judgment? It would be the threshold that minimizes total loss. The essential question then becomes, how can we find this threshold IT*? Two requirements are necessary. First, the discrimination failure rate should be as low as possible. The performance evaluation graph determines the threshold meeting in this condition. The loss depends more on the contents of the failure than on the failure rate. Economic loss is enormous with false negatives leading to the spread of infection than that with false positives that lead to the over-killing of animals. Therefore, the second requirement is that the false-negative rate is small relative to the false positive rate.

#### 4.2.2 Analysis method for locating the threshold for epidemic judgment

**Fig 11:**
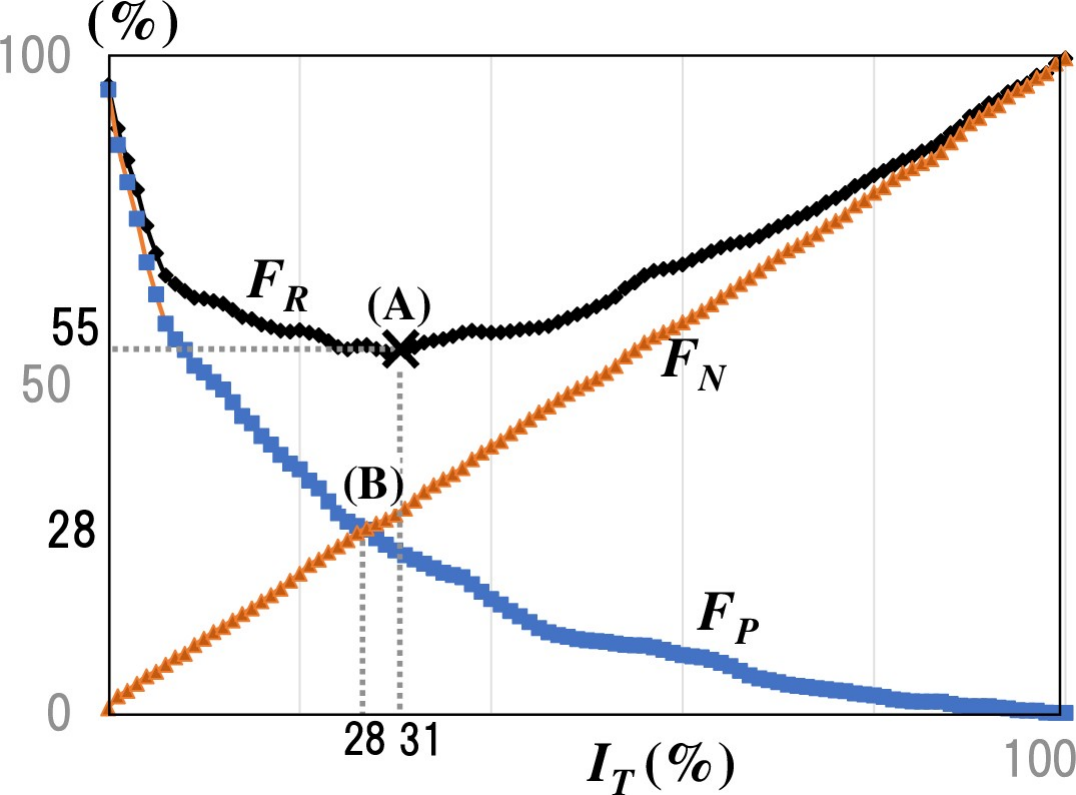
Location of thresholds for making decisions (utilization of the performance evaluation graph). The performance evaluation graph can be regarded as a graph of the estimation failure rate, *F*_*R*_. Point (A) indicates the minimum *F*_*R*_ value. Point (B) marks the intersection of the two components of *F*_*R*_, the false negative rate, *F*_*N*_, and the false positive rate, *F*_*P*_. The graph provides important suggestions regarding the appropriate location of the FMD-VS threshold.

How can we decide where thresholds meet both requirements? The performance evaluation graph in Fig 11 can illustrate. Figure *FR*, in Fig 11, gives the graph of the discrimination failure rate without stratification (Fig 9-Y5,0-<1>) for Y5,0 (*n* = 5, *m* = 0) in Fig 9). We can decompose this discrimination failure rate *FR* into the false-negative rate *FN*, and the false positive rate *FP*.

The two risk occurrence rates intersect because of the trade-off relationship between them. Fig 11-(B) shows the intersection points of the graphs. The intersection is the optimal compromise with the balance of two risks. However, the optimum point for epidemic judgment is not the balance point (i.e., (B), the intersection) since the loss due to a false negative is massive. At a position where false negatives take place well below that of false positives; the highest point should be to the left of the intersection (B). However, with Fig 11, as the point moves to the left, it moves away from the point (A), which marks the estimated overall failure rate minimum, showing that it rises. For this reason, in this case, be desirable to consider values to the left of (B) is seeking a potential threshold value. Based on this observation point (A), which marks the least discrimination failure rate on the left of the intersection (B), it is advantageous.

#### 4.2.3 Results of the analysis

We performed the same analysis on all five classification discrimination performance graphs shown in Section 3 (distance from the center of gravity, animal species, farm size, the combination of the center of gravity and animal species, the combination of the center of gravity and farm size). In each case, we determined the trade-off intersection point (B) and examined its positional relationship to point (A), with the lowest judgment failure rate, and considered whether there was a desirable case (where point (A) is to the left of intersection (B)) for determining the region of possible threshold values. From the results, we found that all three cases that included distances from the center of gravity showed the desirable characteristic.

**Fig 12:**
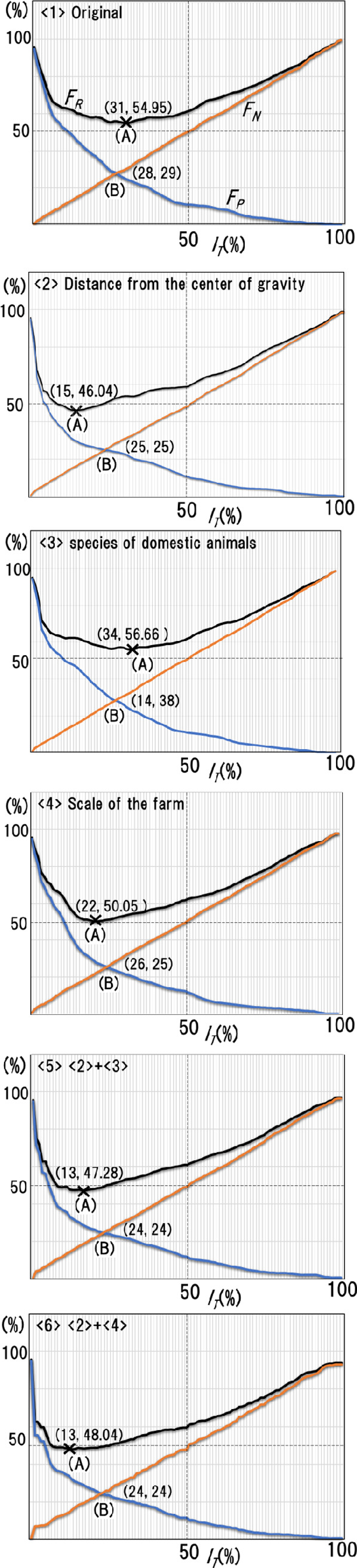
Segment thresholds for decision-making based on various FMD-VS. <1>-<5> show, in effect, the estimation failure rate, *F*_*R*_, for six performance evaluation graphs based on no stratification and five types of stratification (Section 3.1). *F*_*R*_ is broken down into the false negative rate, *F*_*N*_, and the false positive rate, FP. Point (A) marks the minimum value of *F*_*R*_; Point (B) marks the intersection of *F*_*N*_ and *F*_*P*_. In order to best satisfy the decision-making requirements listed in Section 4.2.1 (i.e., (R1), (R2) and (R3)), it would be reasonable to use <2>, <4>, and <5>, with distance from the center of gravity as the basis for segmentation.

Fig 12 shows the contents of this analysis and partial results. In the Figs 1–6 give the analysis results of the six patterns shown in Fig 9. For example, Fig 9-(5) shows the graph obtained by defining “around the time of infection” as ten days before the estimated infection date to the estimated date (*n* = 10, *m* = 0). Fig 12-1 is the analysis result for the no-stratification case; 2 to 6 give the results for each stratification case.

For stratification cases (2, 5, 6), we considered the distance from the center of gravity of the entire infected farm in which the desired graph in which point (A), with the minimum discrimination failure rate, is to the left of the trade-off intersection (B). This feature occurs in cases other than (5) in Fig 9. We could get the same result irrespective of the definition of “around the time of infection.” Based on these findings, we concluded that specifying the area of thresholds for epidemic judgment can be done by using the graph for situations that include consideration of the distance from the center of gravity.

## 5. Conclusion and future work

In this study, we developed the concept of a virtual sensor called FMD-VS, to estimate and show the degree of FMD virus scattering when FMD is suspected. We then proposed a method for using FMD-VS at the time of an outbreak.

In constructing FMD-VS, we simulated a perception from control theory known as a soft sensor, then introduced a formula to estimate and show the scattering situation based on an existing mathematical FMD model. We presented the model of Hayama, which applied the case of the Miyazaki Prefecture, to the well-known Keeling model. We also proposed a performance evaluation method (performance evaluation graph) and a stratification approach to the performance improvement for FMD-VS. We described an effective process for identifying stratification factors. The evaluation experiment with the proposed method resulted in the universal stratification factor that improves the performance of FMD-VS is the center of gravity of the whole infected farm. It changes because of various uncertain influences such as weather, wind direction, and the movement of cars. We considered these useful indicators needless to clarify their contents.

Also, we discussed the concept of a system to support the prevention judgment of the local government staff, aiming to develop and use FMD-VS during the outbreak. We proposed a procedure to narrow the identification of farms on which the animals would need to be destroyed based on the scattering index of each farm calculated by FMD-VS. The overall system comprises two stages: In the first stage, we extract areas of thresholds (thresholds for quarantine judgments) to decide farms where culling would be necessary logically and formally. In the second stage, we choose a single feasible threshold value from among the probabilities. In this paper, we showed the method using the performance evaluation graph from the earlier stage. As a result of our evaluation experiment, we have found that extract the threshold region for the epidemic prevention judgment can be facilitated by using the graph considering the distance from the center of gravity. The second part of the support system involves selecting an appropriate and optimal threshold value within constraints such as the epidemic prevention budget allocated to the infected area. This part of the support system is under development, and we will describe it in our next study.

## Supporting information

S1 Appendix (PDF)
