# Supplementary material for: FMD-VS: A virtual sensor to index FMD virus scattering
Source: PLoS One. 2020 Sep 11;15(9):e0237961. doi: 10.1371/journal.pone.0237961 (PMC7485836; doi:10.1371/journal.pone.0237961)
Supplement: S1 Appendix — (PDF) [file pone.0237961.s001.pdf]

	Area	longitude	latitude	cattle	pigs	onset date	quarantine treatment
1st	都農町 (Tsuno)	131.49667	32.25613	16	0	20/04/2010	21/04/2010
2nd	川南町 (Kawaminami)	131.51446	32.22695	65	0	21/04/2010	24/04/2010
3rd	川南町 (Kawaminami)	131.51557	32.23028	118	0	20/04/2010	25/04/2010
4th	川南町 (Kawaminami)	131.51252	32.22752	65	0	21/04/2010	27/04/2010
5th	川南町 (Kawaminami)	131.51167	32.22724	75	0	22/04/2010	29/04/2010
6th	都農町 (Tsuno)	131.49167	32.25974	42	2	22/04/2010	26/04/2010
7th	川南町 (Kawaminami)	131.51556	32.22722	725	0	24/04/2010	01/05/2010
8th	川南町 (Kawaminami)	131.52862	32.24002	1019	0	27/04/2010	06/05/2010
9th	えびの市 (Ebino cit	130.78444	32.04141	275	0	27/04/2010	01/05/2010
10th	川南町 (Kawaminami)	131.54502	32.21945	0	486	27/04/2010	29/04/2010
11th	川南町 (Kawaminami)	131.51836	32.22833	50	0	28/04/2010	01/05/2010
12th	川南町 (Kawaminami)	131.51056	32.21889	0	1429	29/04/2010	04/05/2010
13th	川南町 (Kawaminami)	131.54444	32.22111	412	3470	30/04/2010	05/05/2010
14th	川南町 (Kawaminami)	131.54722	32.22084	0	299	01/05/2010	06/05/2010
15th	川南町 (Kawaminami)	131.50722	32.19472	424	0	30/04/2010	06/05/2010
16th	川南町 (Kawaminami)	131.54751	32.21419	4	0	02/05/2010	04/05/2010
17th	川南町 (Kawaminami)	131.5525	32.20889	37	0	02/05/2010	06/05/2010
18th	川南町 (Kawaminami)	131.55307	32.21694	0	15747	03/05/2010	18/05/2010
19th	川南町 (Kawaminami)	131.53361	32.22361	0	3010	03/05/2010	12/05/2010
20th	川南町 (Kawaminami)	131.54946	32.21362	0	780	04/05/2010	15/05/2010
21st	川南町 (Kawaminami)	131.55112	32.22223	0	679	04/05/2010	15/05/2010
22nd	えびの市 (Ebino cit	130.78363	32.0453	0	320	04/05/2010	05/05/2010
23rd	川南町 (Kawaminami)	131.55002	32.22306	0	4434	04/05/2010	15/05/2010
24th	川南町 (Kawaminami)	131.54974	32.21278	0	687	04/05/2010	10/05/2010
25th	川南町 (Kawaminami)	131.52946	32.225	0	4221	04/05/2010	18/05/2010
26th	川南町 (Kawaminami)	131.52834	32.22834	0	766	04/05/2010	16/05/2010
27th	川南町 (Kawaminami)	131.54557	32.20724	29	0	04/05/2010	11/05/2010
28th	川南町 (Kawaminami)	131.55028	32.22056	30	0	05/05/2010	15/05/2010
29th	川南町 (Kawaminami)	131.53139	32.22972	0	3488	05/05/2010	15/05/2010
30th	川南町 (Kawaminami)	131.55306	32.22416	50	0	05/05/2010	12/05/2010
31st	川南町 (Kawaminami)	131.54168	32.23333	0	407	05/05/2010	13/05/2010
32nd	川南町 (Kawaminami)	131.52974	32.22695	0	1011	05/05/2010	15/05/2010
33rd	川南町 (Kawaminami)	131.52083	32.2164	0	141	05/05/2010	13/05/2010
34th	川南町 (Kawaminami)	131.52861	32.22529	75	0	05/05/2010	12/05/2010
35th	川南町 (Kawaminami)	131.51166	32.21862	0	2	05/05/2010	10/05/2010
36th	川南町 (Kawaminami)	131.55028	32.21584	46	0	06/05/2010	16/05/2010
37th	川南町 (Kawaminami)	131.56611	32.23027	0	5032	06/05/2010	19/05/2010
38th	川南町 (Kawaminami)	131.53639	32.22779	0	6406	06/05/2010	22/05/2010
39th	川南町 (Kawaminami)	131.52807	32.22057	0	1906	06/05/2010	18/05/2010
40th	川南町 (Kawaminami)	131.51085	32.21751	18	0	06/05/2010	30/05/2010
41st	川南町 (Kawaminami)	131.54778	32.20778	67	0	06/05/2010	18/05/2010
42nd	川南町 (Kawaminami)	131.51474	32.21444	0	640	06/05/2010	15/05/2010
43rd	川南町 (Kawaminami)	131.52056	32.22779	97	0	06/05/2010	15/05/2010
44th	川南町 (Kawaminami)	131.53945	32.22611	0	663	07/05/2010	14/05/2010
45th	川南町 (Kawaminami)	131.53557	32.23001	66	0	07/05/2010	13/05/2010
46th	川南町 (Kawaminami)	131.55501	32.22556	9	0	07/05/2010	13/05/2010
47th	川南町 (Kawaminami)	131.5339	32.23583	691	0	07/05/2010	24/05/2010
48th	都農町 (Tsuno)	131.53751	32.26833	207	0	07/05/2010	13/05/2010
49th	川南町 (Kawaminami)	131.55139	32.21224	186	0	07/05/2010	02/06/2010
50th	川南町 (Kawaminami)	131.52853	32.22474	0	1056	08/05/2010	20/05/2010
51st	川南町 (Kawaminami)	131.54196	32.21307	0	613	08/05/2010	19/05/2010
52nd	川南町 (Kawaminami)	131.515	32.2139	17	0	08/05/2010	19/05/2010
53rd	川南町 (Kawaminami)	131.52835	32.23973	97	0	08/05/2010	17/05/2010
54th	川南町 (Kawaminami)	131.55166	32.2275	126	0	08/05/2010	15/05/2010
55th	川南町 (Kawaminami)	131.51222	32.21694	15	0	08/05/2010	30/05/2010
56th	川南町 (Kawaminami)	131.52529	32.2175	4	0	08/05/2010	01/06/2010
57th	川南町 (Kawaminami)	131.53555	32.20862	0	791	09/05/2010	22/05/2010

58th	川南町 (Kawaminami)	131.55556	32.22724	100	0	09/05/2010	20/05/2010
59th	川南町 (Kawaminami)	131.55085	32.22252	16	0	09/05/2010	23/05/2010
60th	川南町 (Kawaminami)	131.53556	32.23752	0	7903	09/05/2010	30/05/2010
61st	川南町 (Kawaminami)	131.54972	32.21417	9	0	09/05/2010	24/05/2010
62nd	川南町 (Kawaminami)	131.54113	32.23141	15	0	08/05/2010	22/05/2010
63rd	川南町 (Kawaminami)	131.55418	32.21113	100	0	09/05/2010	25/05/2010
64th	川南町 (Kawaminami)	131.54113	32.22084	50	0	09/05/2010	25/05/2010
65th	川南町 (Kawaminami)	131.51446	32.19973	49	0	09/05/2010	17/05/2010
66th	川南町 (Kawaminami)	131.49168	32.19113	645	0	09/05/2010	18/05/2010
67th	川南町 (Kawaminami)	131.54891	32.21335	0	2820	09/05/2010	21/05/2010
68th	えびの市 (Ebino cit	130.78112	32.04168	18	0	10/05/2010	12/05/2010
69th	川南町 (Kawaminami)	131.55334	32.20918	20	0	10/05/2010	15/05/2010
70th	川南町 (Kawaminami)	131.55805	32.22446	0	153	10/05/2010	21/05/2010
71st	川南町 (Kawaminami)	131.52891	32.22502	114	0	10/05/2010	19/05/2010
72nd	都農町 (Tsuno)	131.53891	32.27252	12	0	11/05/2010	14/05/2010
73rd	川南町 (Kawaminami)	131.51389	32.17668	288	0	11/05/2010	16/05/2010
74th	川南町 (Kawaminami)	131.54752	32.22889	0	1098	11/05/2010	21/05/2010
75th	川南町 (Kawaminami)	131.54195	32.22612	0	156	11/05/2010	21/05/2010
76th	川南町 (Kawaminami)	131.5128	32.22362	50	0	11/05/2010	06/06/2010
77th	川南町 (Kawaminami)	131.5403	32.21196	59	0	11/05/2010	19/05/2010
78th	川南町 (Kawaminami)	131.52918	32.22223	107	0	12/05/2010	21/05/2010
79th	川南町 (Kawaminami)	131.51279	32.21028	9	0	12/05/2010	30/05/2010
80th	川南町 (Kawaminami)	131.53752	32.22919	0	180	12/05/2010	24/05/2010
81st	川南町 (Kawaminami)	131.54584	32.24141	0	205	12/05/2010	24/05/2010
82nd	川南町 (Kawaminami)	131.56474	32.23277	0	740	12/05/2010	09/06/2010
83rd	えびの市 (Ebino cit	130.78141	32.05501	46	0	12/05/2010	14/05/2010
84th	川南町 (Kawaminami)	131.51141	32.20724	36	0	12/05/2010	06/06/2010
85th	川南町 (Kawaminami)	131.5478	32.21112	76	0	12/05/2010	01/06/2010
86th	川南町 (Kawaminami)	131.54	32.22418	9	0	12/05/2010	25/05/2010
87th	川南町 (Kawaminami)	131.55501	32.22945	26	0	13/05/2010	26/05/2010
88th	川南町 (Kawaminami)	131.53472	32.23222	35	0	13/05/2010	27/05/2010
89th	川南町 (Kawaminami)	131.56418	32.23445	30	0	13/05/2010	23/05/2010
90th	川南町 (Kawaminami)	131.56473	32.235	13	0	13/05/2010	23/05/2010
91st	川南町 (Kawaminami)	131.54055	32.22501	5	0	13/05/2010	26/05/2010
92nd	川南町 (Kawaminami)	131.5389	32.23083	28	0	13/05/2010	26/05/2010
93rd	川南町 (Kawaminami)	131.56335	32.22947	33	0	13/05/2010	24/05/2010
94th	川南町 (Kawaminami)	131.50724	32.2189	0	941	13/05/2010	30/05/2010
95th	川南町 (Kawaminami)	131.50917	32.19529	148	0	14/05/2010	24/05/2010
96th	川南町 (Kawaminami)	131.54584	32.20778	16	0	14/05/2010	26/05/2010
97th	川南町 (Kawaminami)	131.51307	32.19694	37	0	14/05/2010	24/05/2010
98th	川南町 (Kawaminami)	131.54861	32.21333	7	0	14/05/2010	27/05/2010
99th	川南町 (Kawaminami)	131.55334	32.21278	26	0	14/05/2010	06/06/2010
100th	川南町 (Kawaminami)	131.50474	32.17668	501	0	14/05/2010	07/06/2010
101st	高鍋町 (Takanabe)	131.52695	32.16168	308	0	13/05/2010	01/06/2010
102nd	川南町 (Kawaminami)	131.52917	32.18057	52	0	15/05/2010	30/05/2010
103rd	川南町 (Kawaminami)	131.51083	32.2289	0	1211	15/05/2010	01/06/2010
104th	川南町 (Kawaminami)	131.54307	32.20918	14	0	15/05/2010	27/05/2010
105th	川南町 (Kawaminami)	131.52	32.21388	15	0	15/05/2010	01/06/2010
106th	川南町 (Kawaminami)	131.54667	32.22917	0	594	15/05/2010	27/05/2010
107th	川南町 (Kawaminami)	131.53169	32.23696	53	0	15/05/2010	27/05/2010
108th	都農町 (Tsuno)	131.5514	32.24252	9	0	15/05/2010	18/05/2010
109th	川南町 (Kawaminami)	131.54667	32.2283	0	1112	15/05/2010	29/05/2010
110th	川南町 (Kawaminami)	131.55362	32.20834	32	0	15/05/2010	29/05/2010
111th	高鍋町 (Takanabe)	131.52224	32.16919	218	0	15/05/2010	20/05/2010
112th	川南町 (Kawaminami)	131.51251	32.16585	61	0	15/05/2010	28/05/2010
113th	川南町 (Kawaminami)	131.53	32.17835	15	0	15/05/2010	01/06/2010
114th	川南町 (Kawaminami)	131.50279	32.20945	37	0	15/05/2010	29/05/2010
115th	川南町 (Kawaminami)	131.46555	32.18835	108	0	15/05/2010	05/06/2010
116th	川南町 (Kawaminami)	131.48334	32.19057	0	1227	15/05/2010	02/06/2010

117th	川南町 (Kawaminami)	131.56085	32.21084	41	0	16/05/2010	30/05/2010
118th	川南町 (Kawaminami)	131.51362	32.17806	0	5488	16/05/2010	29/05/2010
119th	新富町 (Shintomi	131.50307	32.10612	22	0	16/05/2010	17/05/2010
120th	川南町 (Kawaminami)	131.55195	32.20278	0	809	16/05/2010	16/06/2010
121st	川南町 (Kawaminami)	131.53085	32.18891	36	0	16/05/2010	07/06/2010
122nd	川南町 (Kawaminami)	131.53916	32.17751	0	8617	16/05/2010	06/06/2010
123rd	川南町 (Kawaminami)	131.52222	32.36806	124	0	16/05/2010	01/06/2010
124th	高鍋町 (Takanabe)	131.53669	32.15111	0	3056	16/05/2010	20/05/2010
125th	川南町 (Kawaminami)	131.53362	32.1775	0	8068	16/05/2010	06/06/2010
126th	川南町 (Kawaminami)	131.48918	32.17333	0	743	16/05/2010	02/06/2010
127th	川南町 (Kawaminami)	131.55502	32.20779	41	0	16/05/2010	02/06/2010
128th	川南町 (Kawaminami)	131.545	32.16474	0	1509	16/05/2010	01/06/2010
129th	川南町 (Kawaminami)	131.53723	32.1689	0	1947	16/05/2010	29/05/2010
130th	高鍋町 (Takanabe)	131.4789	32.11723	247	0	16/05/2010	09/06/2010
131st	新富町 (Shintomi	131.46083	32.12139	243	0	17/05/2010	22/05/2010
132nd	川南町 (Kawaminami)	131.53195	32.17778	75	0	17/05/2010	03/06/2010
133rd	川南町 (Kawaminami)	131.53447	32.17474	49	0	17/05/2010	16/06/2010
134th	川南町 (Kawaminami)	131.51085	32.20946	16	0	17/05/2010	01/06/2010
135th	川南町 (Kawaminami)	131.51085	32.17778	92	0	17/05/2010	04/06/2010
136th	都農町 (Tsuno)	131.56835	32.25335	40	0	17/05/2010	22/05/2010
137th	川南町 (Kawaminami)	131.53945	32.20723	10	0	17/05/2010	02/06/2010
138th	都農町 (Tsuno)	131.54084	32.27418	135	0	17/05/2010	22/05/2010
139th	川南町 (Kawaminami)	131.55611	32.22973	0	782	17/05/2010	30/05/2010
140th	川南町 (Kawaminami)	131.51363	32.15695	0	341	18/05/2010	12/06/2010
141st	新富町 (Shintomi	131.4414	32.12584	800	0	18/05/2010	28/05/2010
142nd	川南町 (Kawaminami)	131.56583	32.23528	0	513	18/05/2010	30/05/2010
143rd	高鍋町 (Takanabe)	131.48361	32.13168	1521	0	18/05/2010	20/06/2010
144th	高鍋町 (Takanabe)	131.46946	32.14752	1316	0	18/05/2010	24/06/2010
145th	川南町 (Kawaminami)	131.53611	32.17446	0	892	18/05/2010	05/06/2010
146th	新富町 (Shintomi	131.43083	32.11194	461	0	18/05/2010	30/05/2010
147th	川南町 (Kawaminami)	131.56252	32.21612	55	0	17/05/2010	10/06/2010
148th	川南町 (Kawaminami)	131.54806	32.21918	7	0	17/05/2010	03/06/2010
149th	川南町 (Kawaminami)	131.56307	32.23055	42	0	17/05/2010	02/06/2010
150th	川南町 (Kawaminami)	131.55279	32.20806	64	0	17/05/2010	03/06/2010
151st	川南町 (Kawaminami)	131.55529	32.23029	49	0	18/05/2010	11/06/2010
152nd	川南町 (Kawaminami)	131.52112	32.18446	38	0	18/05/2010	03/06/2010
153rd	都農町 (Tsuno)	131.58336	32.29667	6	0	18/05/2010	21/05/2010
154th	都農町 (Tsuno)	131.56194	32.24112	16	0	18/05/2010	24/05/2010
155th	川南町 (Kawaminami)	131.53056	32.23446	0	2623	19/05/2010	08/06/2010
156th	川南町 (Kawaminami)	131.50973	32.22862	0	307	19/05/2010	12/06/2010
157th	高鍋町 (Takanabe)	131.48166	32.1325	589	0	19/05/2010	20/06/2010
158th	高鍋町 (Takanabe)	131.48556	32.1325	1050	0	19/05/2010	18/06/2010
159th	川南町 (Kawaminami)	131.5103	32.18169	0	146	19/05/2010	04/06/2010
160th	川南町 (Kawaminami)	131.56002	32.23529	0	690	16/05/2010	18/06/2010
161st	川南町 (Kawaminami)	131.5228	32.17557	231	0	20/05/2010	09/06/2010
162nd	川南町 (Kawaminami)	131.53806	32.16669	8	0	20/05/2010	29/05/2010
163rd	木城町 (kijo)	131.4575	32.14306	884	0	20/05/2010	28/05/2010
164th	高鍋町 (Takanabe)	131.50556	32.12779	3	0	20/05/2010	31/05/2010
165th	川南町 (Kawaminami)	131.53223	32.17862	54	0	20/05/2010	04/06/2010
166th	西都市 (Saito city)	131.435	32.13111	200	0	20/05/2010	24/05/2010
167th	高鍋町 (Takanabe)	131.51807	32.16917	9	0	20/05/2010	03/06/2010
168th	新富町 (Shintomi	131.48835	32.11389	165	0	20/05/2010	31/05/2010
169th	新富町 (Shintomi	131.485	32.09974	26	0	20/05/2010	26/05/2010
170th	高鍋町 (Takanabe)	131.51613	32.12029	115	0	20/05/2010	01/06/2010
171st	西都市尾八重 (Saito	131.32028	32.27391	6	0	19/05/2010	22/05/2010
172nd	木城町 (kijo)	131.48502	32.165	0	269	20/05/2010	01/06/2010
173rd	川南町 (Kawaminami)	131.54972	32.23279	62	0	20/05/2010	07/06/2010
174th	川南町 (Kawaminami)	131.51724	32.21444	32	0	21/05/2010	29/05/2010
175th	川南町 (Kawaminami)	131.51723	32.16641	4	0	21/05/2010	29/05/2010

176th	川南町	(Kawaminami)	131.5478	32.17891	66	0	21/05/2010	06/06/2010
177th	高鍋町	(Takanabe)	131.53362	32.15083	118	0	21/05/2010	06/06/2010
178th	高鍋町	(Takanabe)	131.53474	32.15055	0	1685	21/05/2010	31/05/2010
179th	川南町	(Kawaminami)	131.53085	32.17807	22	0	21/05/2010	07/06/2010
180th	川南町	(Kawaminami)	131.49613	32.19613	60	0	21/05/2010	08/06/2010
181st	川南町	(Kawaminami)	131.53973	32.17474	0	936	21/05/2010	18/06/2010
182nd	都農町	(Tsuno)	131.53029	32.27	242	0	21/05/2010	10/06/2010
183rd	新富町	(Shintomi	131.43779	32.10722	255	0	21/05/2010	09/06/2010
184th	新富町	(Shintomi	131.44306	32.12583	84	0	21/05/2010	28/05/2010
185th	川南町	(Kawaminami)	131.53972	32.17083	15	0	21/05/2010	04/06/2010
186th	川南町	(Kawaminami)	131.5414	32.23002	0	0	21/05/2010	27/05/2010
187th	川南町	(Kawaminami)	131.56279	32.22834	6	0	21/05/2010	05/06/2010
188th	西都市	(Saito city)	131.4375	32.13556	157	0	22/05/2010	26/05/2010
189th	川南町	(Kawaminami)	131.55919	32.2378	0	321	22/05/2010	02/06/2010
190th	川南町	(Kawaminami)	131.52474	32.18083	0	355	22/05/2010	27/05/2010
191st	川南町	(Kawaminami)	131.48836	32.17111	0	5192	22/05/2010	01/06/2010
192nd	川南町	(Kawaminami)	131.51002	32.21473	106	959	22/05/2010	02/06/2010
193rd	川南町	(Kawaminami)	131.53752	32.17446	24	0	22/05/2010	16/06/2010
194th	川南町	(Kawaminami)	131.55306	32.20722	4	0	22/05/2010	09/06/2010
195th	新富町	(Shintomi	131.44389	32.12834	441	0	22/05/2010	01/06/2010
196th	都農町	(Tsuno)	131.54333	32.23502	3	0	22/05/2010	26/05/2010
197th	高鍋町	(Takanabe)	131.49584	32.12889	53	281	23/05/2010	26/05/2010
198th	川南町	(Kawaminami)	131.53667	32.21002	18	0	23/05/2010	07/06/2010
199th	川南町	(Kawaminami)	131.56306	32.23639	26	0	23/05/2010	07/06/2010
200th	新富町	(Shintomi	131.48918	32.11113	179	0	23/05/2010	03/06/2010
201st	川南町	(Kawaminami)	131.51585	32.1953	24	0	23/05/2010	09/06/2010
202nd	新富町	(Shintomi	131.48889	32.11029	107	0	23/05/2010	04/06/2010
203rd	都農町	(Tsuno)	131.5328	32.26919	59	0	24/05/2010	27/05/2010
204th	川南町	(Kawaminami)	131.5425	32.16391	40	0	24/05/2010	01/06/2010
205th	木城町	(kijo)	131.45389	32.17334	0	2119	24/05/2010	03/06/2010
206th	川南町	(Kawaminami)	131.54417	32.17334	9	0	24/05/2010	28/05/2010
207th	川南町	(Kawaminami)	131.54695	32.20473	14	0	24/05/2010	09/06/2010
208th	川南町	(Kawaminami)	131.54083	32.1614	157	0	24/05/2010	08/06/2010
209th	川南町	(Kawaminami)	131.49973	32.19529	7	0	24/05/2010	08/06/2010
210th	川南町	(Kawaminami)	131.51252	32.22446	96	0	24/05/2010	06/06/2010
211th	川南町	(Kawaminami)	131.54889	32.20416	32	0	25/05/2010	09/06/2010
212th	都農町	(Tsuno)	131.535	32.26889	118	0	25/05/2010	29/05/2010
213th	高鍋町	(Takanabe)	131.5364	32.15028	0	525	23/05/2010	02/06/2010
214th	川南町	(Kawaminami)	131.5325	32.18279	0	1139	25/05/2010	07/06/2010
215th	川南町	(Kawaminami)	131.55807	32.23472	0	80	25/05/2010	10/06/2010
216th	高鍋町	(Takanabe)	131.47695	32.1314	2317	0	25/05/2010	24/06/2010
217th	川南町	(Kawaminami)	131.49973	32.19307	26	0	25/05/2010	07/06/2010
218th	川南町	(Kawaminami)	131.54861	32.22834	16	0	25/05/2010	02/06/2010
219th	川南町	(Kawaminami)	131.52556	32.1814	15	0	25/05/2010	01/06/2010
220th	都農町	(Tsuno)	131.56861	32.24667	10	0	26/05/2010	29/05/2010
221st	川南町	(Kawaminami)	131.53057	32.17361	21	0	26/05/2010	10/06/2010
222nd	高鍋町	(Takanabe)	131.45307	32.13779	0	2716	26/05/2010	08/06/2010
223rd	川南町	(Kawaminami)	131.53196	32.17697	45	0	27/05/2010	04/06/2010
224th	川南町	(Kawaminami)	131.5303	32.19029	18	0	27/05/2010	03/06/2010
225th	川南町	(Kawaminami)	131.53418	32.23335	0	576	27/05/2010	07/06/2010
226th	川南町	(Kawaminami)	131.50835	32.21916	0	279	28/05/2010	11/06/2010
227th	高鍋町	(Takanabe)	131.46	32.13361	3957	0	28/05/2010	28/06/2010
228th	高鍋町	(Takanabe)	131.47666	32.11833	1018	0	28/05/2010	22/06/2010
229th	高鍋町	(Takanabe)	131.46252	32.14194	620	0	28/05/2010	21/06/2010
230th	都農町	(Tsuno)	131.54141	32.27555	34	0	28/05/2010	01/06/2010
231st	川南町	(Kawaminami)	131.50279	32.19472	46	0	28/05/2010	12/06/2010
232nd	川南町	(Kawaminami)	131.53806	32.1753	48	0	28/05/2010	16/06/2010
233rd	新富町	(Shintomi	131.46473	32.1203	353	0	28/05/2010	07/06/2010
234th	川南町	(Kawaminami)	131.50944	32.23333	3	0	29/05/2010	06/06/2010

235th	西都市	(Saito city)	131.43308	32.12944	873	0	29/05/2010	12/06/2010
236th	都農町	(Tsuno)	131.56833	32.24474	4	0	29/05/2010	01/06/2010
237th	新富町	(Shintomi	131.48945	32.08694	35	0	29/05/2010	04/06/2010
238th	都農町	(Tsuno)	131.58446	32.29863	54	0	29/05/2010	01/06/2010
239th	川南町	(Kawaminami)	131.5353	32.23444	39	0	29/05/2010	10/06/2010
240th	高鍋町	(Takanabe)	131.5325	32.15363	43	0	29/05/2010	12/06/2010
241st	川南町	(Kawaminami)	131.51362	32.20722	23	0	30/05/2010	08/06/2010
242nd	川南町	(Kawaminami)	131.50667	32.19917	87	0	30/05/2010	12/06/2010
243rd	都農町	(Tsuno)	131.53752	32.27195	253	0	30/05/2010	04/06/2010
244th	都農町	(Tsuno)	131.5339	32.26918	28	0	30/05/2010	04/06/2010
245th	都農町	(Tsuno)	131.54084	32.26473	12	0	30/05/2010	05/06/2010
246th	川南町	(Kawaminami)	131.49336	32.20527	55	0	30/05/2010	11/06/2010
247th	都農町	(Tsuno)	131.56444	32.2525	12	0	30/05/2010	08/06/2010
248th	都農町	(Tsuno)	131.51472	32.26752	0	4680	30/05/2010	07/06/2010
249th	都農町	(Tsuno)	131.55808	32.25974	5	0	30/05/2010	08/06/2010
250th	高鍋町	(Takanabe)	131.48416	32.11474	43	0	30/05/2010	14/06/2010
251st	都農町	(Tsuno)	131.53724	32.28889	26	0	31/05/2010	09/06/2010
252nd	川南町	(Kawaminami)	131.50695	32.1964	291	0	31/05/2010	15/06/2010
253rd	西都市	(Saito city)	131.43277	32.12946	779	0	31/05/2010	07/06/2010
254th	川南町	(Kawaminami)	131.51306	32.21333	56	0	01/06/2010	10/06/2010
255th	都農町	(Tsuno)	131.52918	32.26555	0	775	01/06/2010	07/06/2010
256th	高鍋町	(Takanabe)	131.46722	32.12251	0	2014	01/06/2010	12/06/2010
257th	都農町	(Tsuno)	131.53667	32.27391	119	0	01/06/2010	07/06/2010
258th	川南町	(Kawaminami)	131.53668	32.16028	0	4815	01/06/2010	24/06/2010
259th	川南町	(Kawaminami)	131.53611	32.17918	0	790	01/06/2010	19/06/2010
260th	川南町	(Kawaminami)	131.53002	32.18085	26	0	30/05/2010	15/06/2010
261st	川南町	(Kawaminami)	131.51695	32.23278	0	597	02/06/2010	11/06/2010
262nd	都農町	(Tsuno)	131.5314	32.27334	82	0	02/06/2010	05/06/2010
263rd	川南町	(Kawaminami)	131.5125	32.21001	41	0	02/06/2010	18/06/2010
264th	都農町	(Tsuno)	131.53279	32.2625	6	0	02/06/2010	06/06/2010
265th	新富町	(Shintomi	131.45778	32.11835	22	0	02/06/2010	09/06/2010
266th	高鍋町	(Takanabe)	131.48279	32.1514	62	0	02/06/2010	15/06/2010
267th	川南町	(Kawaminami)	131.495	32.19918	12	0	03/06/2010	08/06/2010
268th	川南町	(Kawaminami)	131.52946	32.23502	0	616	03/06/2010	11/06/2010
269th	都農町	(Tsuno)	131.5303	32.27555	80	0	03/06/2010	06/06/2010
270th	川南町	(Kawaminami)	131.48085	32.18194	0	276	02/06/2010	11/06/2010
271st	新富町	(Shintomi	131.43639	32.10668	43	0	04/06/2010	10/06/2010
272nd	川南町	(Kawaminami)	131.51028	32.12027	63	0	04/06/2010	18/06/2010
273rd	新富町	(Shintomi	131.48835	32.10085	372	0	05/06/2010	11/06/2010
274th	木城町	(kijo)	131.47696	32.14835	0	655	05/06/2010	10/06/2010
275th	都農町	(Tsuno)	131.5264	32.27668	25	0	05/06/2010	09/06/2010
276th	川南町	(Kawaminami)	131.55195	32.20807	50	0	06/06/2010	12/06/2010
277th	川南町	(Kawaminami)	131.49307	32.20306	0	2605	07/06/2010	19/06/2010
278th	川南町	(Kawaminami)	131.48168	32.19391	0	1309	07/06/2010	21/06/2010
279th	高鍋町	(Takanabe)	131.47612	32.10861	332	0	08/06/2010	23/06/2010
280th	都城市	(Miyakonojo	131.1314	31.90197	208	0	09/06/2010	12/06/2010
281st	木城町	(kijo)	131.46335	32.18473	0	1760	09/06/2010	15/06/2010
282nd	川南町	(Kawaminami)	131.52001	32.23334	0	595	09/06/2010	22/06/2010
283rd	西都市	(Saito city)	131.35639	32.08808	550	0	10/06/2010	12/06/2010
284th	日向市	(Hyuga city)	131.6175	32.34945	349	0	10/06/2010	12/06/2010
285th	宮崎市	(Miyazaki ci	131.3839	31.95028	0	1325	10/06/2010	12/06/2010
286th	川南町	(Kawaminami)	131.49278	32.20419	74	0	10/06/2010	14/06/2010
287th	西都市	(Saito city)	131.41778	32.13445	1351	0	10/06/2010	25/06/2010
288th	新富町	(Shintomi	131.49418	32.09641	0	3304	11/06/2010	22/06/2010
289th	西都市	(Saito city)	131.35557	32.09278	33	0	13/06/2010	14/06/2010
290th	国富町	(Kumitomi)	131.3675	31.98889	234	0	16/06/2010	18/06/2010
291st	宮崎市	(Miyazaki ci	131.39417	31.94252	38	0	18/06/2010	19/06/2010
292nd	宮崎市	(Miyazaki ci	131.38945	31.9464	16	0	04/07/2010	05/07/2010
